# A framework for assessing urban carrying capacity and its impact on water and sanitation service delivery in a hill city

**DOI:** 10.1007/s43621-026-02959-7

**Published:** 2026-05-12

**Authors:** Vanshika Bajaj, Tanya Ahmed, Anant Mitra, Shantanu Padhi

**Affiliations:** National Institute of Urban Affairs (NIUA), Delhi, India

**Keywords:** Carrying capacity, Water, Sanitation, Wastewater, Tourism

## Abstract

In March 2021, the National Green Tribunal of India (NGT), directed states to assess the carrying capacities of Indian hill cities to balance tourism and ecological sustainability. This mandate was the first stepping stone to restrict unplanned development and regulate tourist influx that can be sustained by the cities. Nainital, a major tourist destination in Uttarakhand, faces immense pressure on its urban infrastructure and service delivery, especially in the context of water and sanitation, due to the rising tourist influx. However, India lacks a national-level framework for assessing and managing the carrying capacity of the urban environment. This study, conducted by National Institute of Urban Affairs (NIUA), develops a comprehensive framework for assessing carrying capacity by using key indicators, correction factor-based calculations, and temporal analysis. A mixed-method approach, including a systematic literature review, key informant interviews (KIIs), and focus group discussions (FGDs), was employed. According to the URDPFI Guidelines, while the city could theoretically accommodate 8,34,184 persons per day based on physical carrying capacity, the number reduced to 149,230 based on real carrying capacity, while the effective carrying capacity is limited to approximately 8,422 due to resource and service delivery constraints. The research offers policymakers a framework for sustainable water and sanitation management, aiding urban planning in ecologically sensitive areas.

**Supplementary Information:**

The online version contains supplementary material available at https://doi.org/10.1007/s43621-026-02959-7.

## Introduction

India’s urban population is projected to surpass 600 million, approximately 40 per cent of the total population by 2036, rising from 31 per cent in 2011 [1]. This urban expansion is being witnessed across the country, with significant ecological implications for the Indian Himalayan Region (IHR), characterized by steep relief, unstable geology and a sensitive climate, which renders it highly vulnerable to both natural hazards and anthropogenic disturbances [2].The IHR comprises nearly 17 per cent of the geographical area of India (0.537 million km^2^), with 52 million population and extends over 2500 km between the Indus and the Brahmaputra river systems [3]. Historically, the Indian Himalayan region has been a centre of spiritualism. Today, it attracts a diverse range of visitors, including those interested in adventure, nature, and cultural tourism [4]

Increased tourism supplemented by rapid, unplanned, and unregulated urbanization has been observed across the IHR [5]. As a result, increased population pressures have been associated with a rise in natural hazards, including flash floods, landslides, erratic rainfall, earthquakes, and notable reductions in glacial and green cover. Between 2013 and 2022, the IHR experienced 44 per cent of India’s natural disasters, including 192 incidents of floods, landslides, and thunderstorms [6]. In Uttarakhand, a key tourist destination within the IHR, tourist arrivals were recorded at 70 million in 2023, with a projected rise to 84 million in 2024 [7] even as the state experienced four major disaster events over the past decade, resulting in more than 6000 deaths and several hundred people reported missing (Kedarnath cloudburst, 2013; Chamoli landslide, 2021; Joshimath land subsidence, 2023; Dharali cloudburst, 2025) [8, 9].

While tourism-driven economic growth is being pursued, it raises serious concerns regarding Uttarakhand’s ecological fragility, biodiversity and stress on natural resources. The resultant surge in tourist influx and resident population in the state exacerbates pressures on resources, leading to ad-hoc infrastructure planning, depleted resources and chronic service deficits. This underscores the need for concerted action to balance ecological imperatives, tourism demands, and service delivery infrastructure by integrating carrying capacity considerations, in order to sustain the healthy functioning of cities across the IHR.

Complementing the underscored concerns for cities in the IHR, in March 2022, the National Green Tribunal of India (NGT) under (OA No. 178/2022), directed IHR states to assess the carrying capacities in their respective states to balance tourism and ecological sustainability, to restrict unplanned development and to regulate the population that can be sustained by the cities in the region [10]. While existing scholarships have examined urban carrying capacity by defining thresholds based on environmental, ecological, and social indicators for sustainable city development [11–15] their scope remains limited. These assessments do not adequately account for urban service delivery systems, specifically water and sanitation–services that are directly and severely affected by rapid population growth and tourist influx. This oversight is critical, given that water supply and sanitation are recognized as basic human rights in accordance with Sustainable Development Goal 6 (SDG 6). Addressing this gap, this study advances the existing literature by evaluating carrying capacity through the lens of its implications for urban service delivery, employing a context-specific, city-level assessment framework that captures capacity constraints across the IHR. Given that delivery of urban services are core responsibilities of city administration, this research responds to an identified need by proposing a replicable and scalable framework that aligns water and sanitation infrastructure planning with increasing tourism pressures. Applied to the city of Nainital, Uttarakhand, the framework and its findings are intended to support city-level decision-makers in making informed planning choices toward water, sanitation and tourism infrastructure that adequately serves both resident and tourist populations.

### Objectives

The objectives of the research study are outlined below.

- Evaluate the physical, real and effective carrying capacities of the city of Nainital
- Assessment of existing service delivery infrastructure capacities (water supply, sanitation, wastewater management) and tourism infrastructure (lodging facilities, parking bays)
- Conduct temporal analysis of key assessment indicators with respect to tourism influx for the study area
- Develop a framework for assessing carrying capacity in urban local bodies (ULBs) across the IHR

### Rationale for the selection of Nainital as a pilot city

Nainital was selected as a pilot city due to its strategic relevance in the IHR and high tourist footfall, which has placed significant strain on water and sanitation infrastructure. The city was proposed by the Urban Development Directorate (UDD) following an MoU signed with NIUA to support improved urban sanitation service delivery in Uttarakhand. Established in the mid-nineteenth century as a colonial hill station, Nainital evolved around the Naini Lake and inherited a fragile urban form shaped by steep topography. Over time, its historical role as an administrative and institutional centre reinforced dense development patterns in environmentally sensitive zones. The city’s status as a state judicial capital, an educational hub, and a high tourist footfall city amid a disaster-prone area highlighted the need for sustainable planning. The availability of relevant data further enabled comprehensive research to be undertaken. As a prominent tourist destination, Nainital faces increasing pressure on its natural resources and urban infrastructure while being located in Seismic Zone IV, making it highly susceptible to earthquakes [16]. Furthermore, human-induced environmental degradation has exacerbated the city’s vulnerability to natural hazards; between 2005 and 2010, an average of 35,602 m^2^ of forest land was encroached annually, intensifying risks such as landslides [16]. Given these compounded risks, an assessment of the city’s carrying capacity and infrastructure limits is essential to inform strategies that balance tourism growth with ecological sustainability.

## Theoretical background and literature review

### Environmental and tourism dynamics shaping carrying capacity in the IHR:

**Fig. 1:**
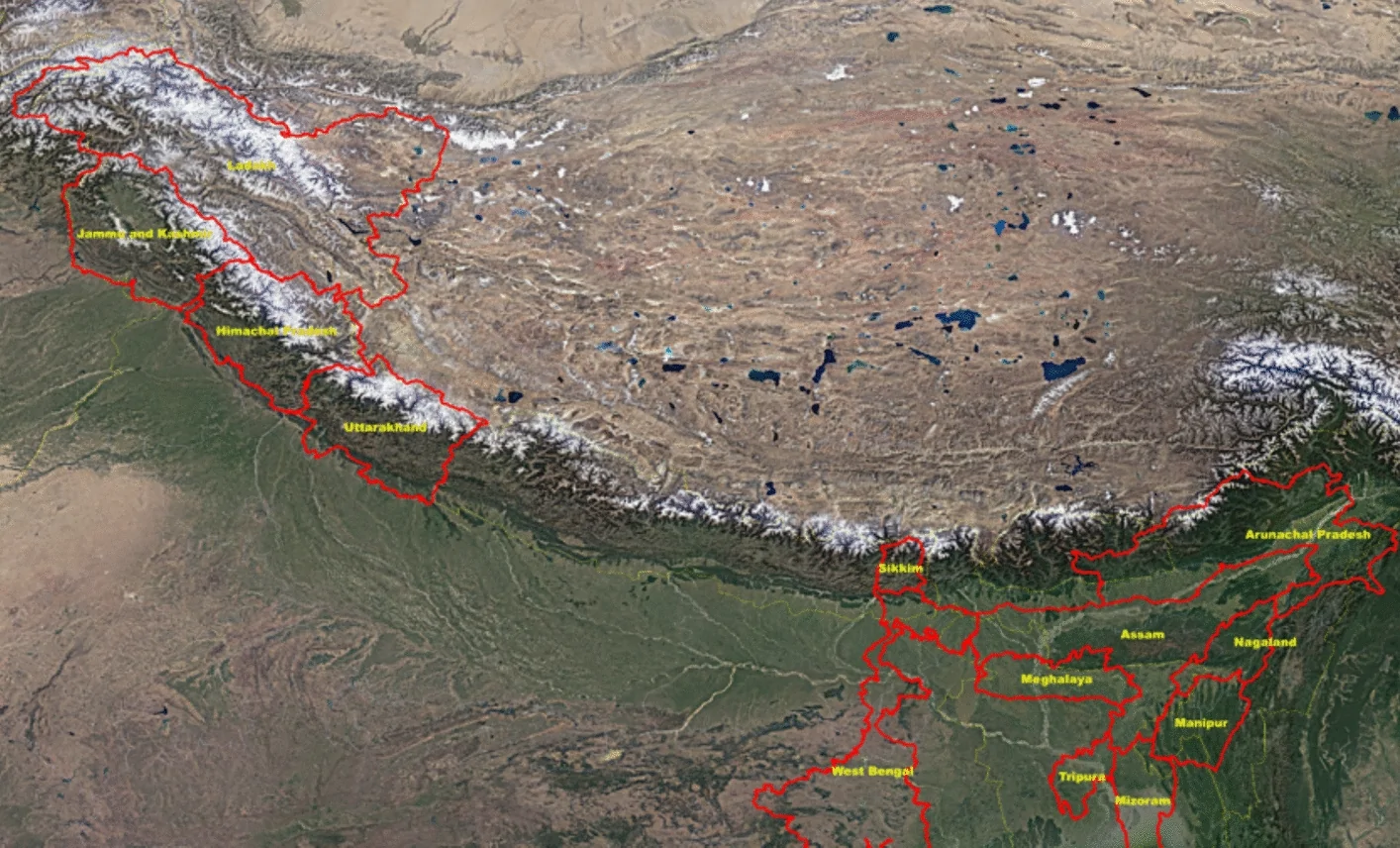
Snow cover in IHR, 31 Dec’1984, Source: Google Earth Pro Historical Imagery

**Fig. 2:**
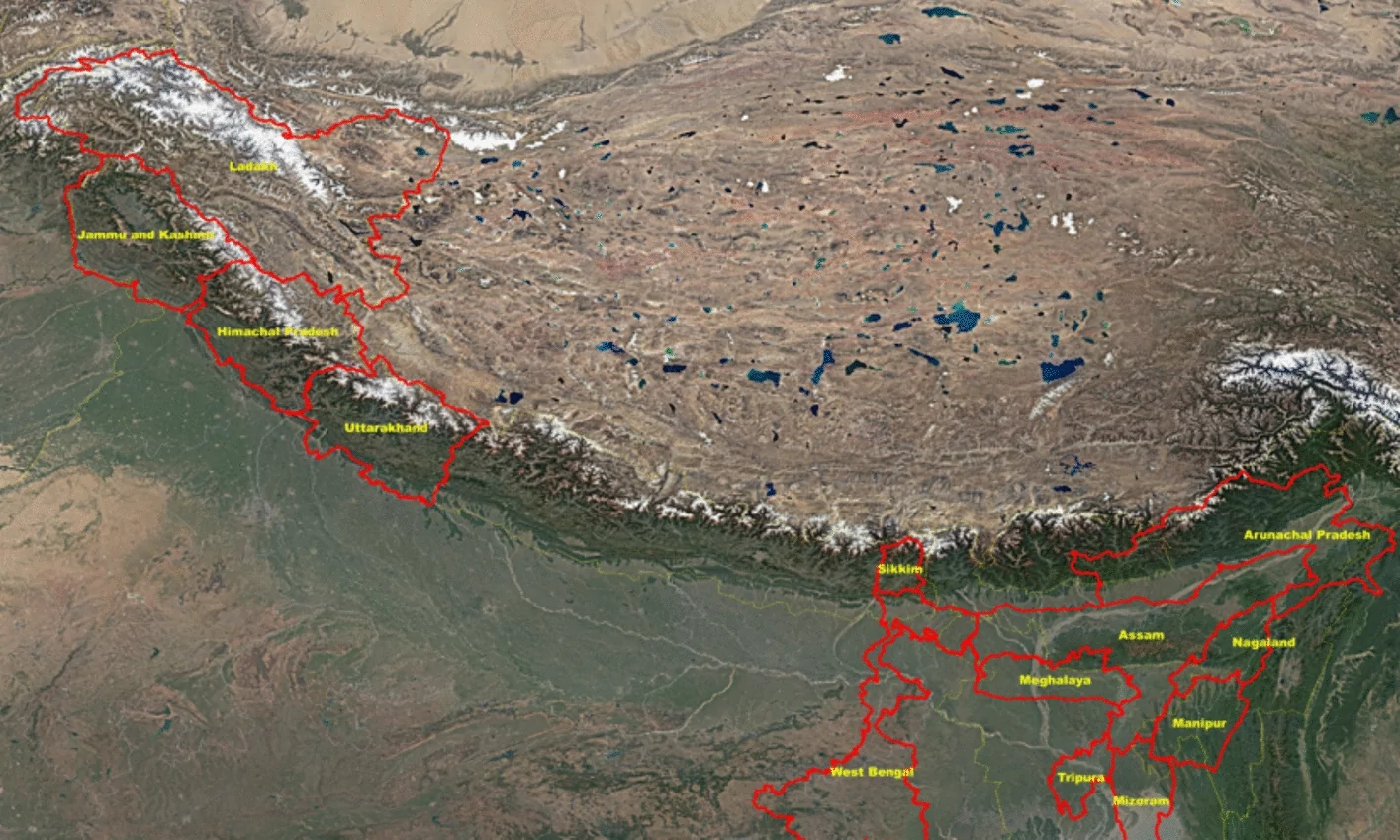
Snow cover in IHR, 1 January 2021, Source: Google Earth Pro Historical Imagery

A 40 per cent reduction in the Himalayan glacier cover over the last four decades (since 1980) was reported in 2021 [17]. Furthermore, a notable decrease can be visually observed through Figs. 1 and 2 below (which present google earth images of the snow cover across the IHR in 1984 and in 2021 respectively). The forest cover in the IHR has decreased by 30.42 per cent and the built-up has increased by 61 per cent in the IHR between 1985 and 2015 [18]. The area statistics based on the Land Use Land Cover (LULC) dataset using satellite data indicate that forest cover was reduced considerably, by 54.71 per cent in Jammu and Kashmir, 44 per cent in Himachal Pradesh, 11.64 percent in Uttarakhand, 5.85 per cent in Sikkim, and 11.23 per cent in Arunachal Pradesh, from 1985 to 2015. The built-up area expanded rapidly from 1985 to 2015, by 28.6 per cent in Sikkim to 30.5 per cent in Uttarakhand [18]. Vital resources such as water and land have been strained, and access to basic services like electricity, water supply, and sanitation has been disrupted due to environmental changes. Increased regional accessibility, globalization, and aggressive marketing have been credited with driving a surge in tourism, further burdening limited infrastructure.

The catastrophic event of land subsidence that occurred in Joshimath town, Uttarakhand, in 2023, when a torrent of water surged down the town’s lower slopes, leading to structural damage, including cracks in buildings, roads, and agricultural fields. The rapid urbanization of the town, with minimal regard for its carrying capacity, was one of the many factors attributed to this vulnerability, as the town’s population grew from approximately 16,709 in 2011 [19] to nearly 22,900 as of 2023 [20]. The town’s infrastructure and environment were further strained by an influx of nearly 5,00,000 tourists during the 2019 season–a 14.6 per cent rise from 2018–and by deforestation linked to ropeway construction to the Auli ski slopes [21]. In the aftermath of the subsidence, parts of Joshimath have been rendered virtually uninhabitable, with widespread cracks and ground deformation. As a precautionary measure, approximately 1000 residents were evacuated from high-risk zones and structurally compromised buildings, reflecting the severity of the hazard and the town’s diminishing capacity to safely support its population [22]. These events also underscore the broader vulnerability of Uttarakhand–a state long recognized for its high exposure to natural hazards [23].

Beyond episodic disasters such as the Joshimath subsidence, districts across Uttarakhand–including Nainital –are experiencing systemic environmental stress driven by changing climatic conditions. In Nainital district, fluctuations in rainfall patterns have contributed to recurrent flash floods, degradation of vegetation health, increased surface run-off and soil erosion, reduced surface moisture, and a higher frequency of landslides [24, 25]. At the same time, rising mean annual temperatures have led to the drying of lakes and increasing stress on water availability, particularly during the summer season, directly affecting urban water supply systems [26–29]. Collectively, these findings underscore the need to systematically examine key climatic indicators over the years–specifically rainfall variability, temperature trends, and landslide occurrence–at the scale of Nainital city, where climate stressors intersect with rapid urbanisation and growing tourism-related pressures, further constraining local carrying capacity and resilience.

Existing literature highlighted that between 2002 and 2013, an average annual tourist growth rate of 28.78 per cent was recorded in the IHR, and by 2019, tourist numbers had exceeded the local population by 1.6 times [24]. Over the past three decades, tourism in the IHR has been continuously expanded and diversified, aligning with a projected average annual growth of 7.9 per cent from 2013 to 2023 [4]. Although tourism is a state subject, the central government, recognizing the sector’s significant contribution to national GDP, has been providing targeted financial support to sustain and accelerate tourism-led growth. A total allocation of approximately ₹3800 crores (423 million USD) has been invested across ten States and two Union Territories (UTs) under central missions such as *Swadesh Darshan 2.0, the Pilgrimage Rejuvenation and Spiritual Augmentation Drive (PRASAD), and the Special Assistance to States for Capital Investment (SASCI) Scheme* [30]. These investments, aimed at strengthening tourism-related infrastructure, are expected to attract a higher influx of visitors, which in turn may intensify pressures on ecology and public service delivery systems. In response, the state of Uttarakhand launched a tourism policy in 2023 to attract over ₹20,000 crore (2.4 billion USD) in investments and implement 100 public–private partnerships (PPPs) for tourism-related projects by 2030 [31].

### Evolution and application of carrying capacity frameworks in tourism and urban contexts

The concept of *Carrying Capacity* was first introduced by Thomas Malthus in 1798, highlighting the Earth’s finite ability to support human populations. In tourism, the United Nations World Tourism Organization (UNWTO) defined Tourism Carrying Capacity (TCC) in the 1980s as the maximum number of individuals who can visit a destination without causing harm to its environment or reducing visitor satisfaction [32]. Similarly, Mc Neely and Thorsell [33] framed it as the maximum visitor level that maintains resource integrity and visitor satisfaction. Mathieson A [34], emphasized preserving recreational experience quality, while Cifuentes in 1992 and 1999 [35] introduced bio-physical and social limits within environmental carrying capacity. Cifuentes further conceptualized carrying capacity through three key dimensions: Physical Carrying Capacity (PCC), Real Carrying Capacity (RCC), and Effective Carrying Capacity (ECC) [35]. PCC is defined as maximum number of visits that can be made to a site with a defined space, in a given time, while RCC (also known as Ecological Carrying capacity) is the maximum allowable number of visitors within a given area, once the reductive/corrective factors (Cf) derived from the particular characteristics of the site in form of ecological conditions such as slope, rainfall etc., has been applied to the PCC. ECC is defined as the maximum number of people that an area can sustain, given the management capacity such as tourism infrastructure and essential services that are available at a particular place.

Following the National Green Tribunal’s 2020 directive mandating the assessment of carrying capacity for at least one Eco-Sensitive Zone (ESZ) in every state, the Central Pollution Control Board (CPCB) [11] undertook a structured Environmental Carrying Capacity study for the ESZ around Sanjay Gandhi National Park (SGNP), Mumbai. Eco-Sensitive Zones (ESZs) or Ecologically Fragile Areas (EFAs) are areas around Protected Areas, National Parks and Wildlife Sanctuaries in India, notified by the Ministry of Environment, Forests and Climate Change (MoEF&CC) to create “shock absorbers” to the protected areas by regulating and managing the activities around such areas. The study employed a two-stage approach. Firstly, delineating the ESZ using remote sensing to map terrain, land use, vegetation, water bodies, and human activities; and then evaluating sector-wise carrying capacity across air, water, noise, tourism, land, and habitat quality. The methodology combined simplified indicators, such as utilization ratios for air and water, tourism carbon carrying capacity, and noise exceedance analysis, with advanced geospatial tools, allowing the study to balance ecological resilience and ecological pressure factors to derive a composite Environmental Carrying Capacity score. The study presented a replicable framework, notwithstanding its dependence on secondary data. Although the research extensively establishes interlinkages among ecology, tourism, air pollution, and the urban heat island effect, its scope remains constrained. Specifically, the focus is limited to a delineated green area and does not integrate the urban infrastructure and service delivery. This pioneer study conducted by CPCB was shared with all the states as a reference framework for conducting carrying capacity assessments for protected areas.

Later in year 2023, after the Joshimath land subsidence disaster, NGT directed the state of Uttarakhand to undertake an urgent assessment of Mussoorie, based on the similar vulnerability to unplanned construction and infrastructure stress [12]. The carrying capacity assessment employed a broad descriptive approach using secondary data on geology, land use, water resources, waste management systems, and tourism influx to identify infrastructural and ecological pressures. The approach was largely advisory and diagnostic based on existing designed capacity of current infrastructure. It lacked a defined methodology and failed to integrate the crucial interlinkages among ecology and infrastructure, which are essential for a systematic, forward-looking planning approach.

Another study from Uttarakhand [15], focusing on the Chardham Yatra–Badrinath, Kedarnath, Gangotri and Yamunotri, where tourist numbers have grown from 1 million annually in the early 2000s to over 3 million recently, with a record 5 million visitors in 2023, adopts a spatial, GIS-based approach to assess carrying capacity. The Multi-Criteria Decision Analysis (MCDA) framework overlayed 21 sub-indicators based on infrastructure, terrain, LULC, accessibility, and environmental sensitivity to produce suitability and capacity maps. Although the Cifuentes model was referenced, the study did not apply correction factors or estimate sectoral thresholds for water supply, waste management, service delivery, or seasonal population fluctuation. As a result, the outputs reflected land-suitability zoning rather than quantifiable carrying capacity, limiting its usefulness for urban-scale, infrastructure-oriented assessments.

The study conducted in Leh [14] was conducted using modified Cifuentes and Ceballos formulae, calculating PCC based on recreational area and tourist density while excluding the resident population in calculations. They clubbed and directly estimated effective capacity after PCC, integrating limiting factors such as weather, transport, accommodation, and waste management, rather than separately deriving RCC and ECC. However, the study was constrained by static datasets, the absence of seasonal variability, limited attention to terrain and glacier-fed water fluctuations, and the omission of essential services like water supply and sanitation. Thus, while it provided a useful sectoral snapshot, it did not fully capture critical infrastructure and service-delivery constraints.

Similarly, the study conducted in Kinnaur, Himachal Pradesh [13] adopted the Cifuentes model to calculate PCC, RCC, and ECC across selected tourist spots, using a set of natural (slope, rainfall, snowfall, frost, temperature) and man-made (accommodation, transportation) correction factors. While the approach followed the standard three-tier Cifuentes framework, its emphasis remained on site-specific correction factors rather than city-level infrastructure systems. Importantly, the estimation of ECC relied heavily on a user-satisfaction survey to score management capacities of tourism sites, such as accessibility, amenities, waste bins, personnel, and safety, rather than quantitative service-delivery metrics. While the study is useful for destination-level conservation, it did not evaluate broader infrastructure capacities, seasonal variations, or municipal service loads central to a city-level carrying capacity framework.

Building on the gaps identified across the reviewed literature–those summarized in the limitation/existing gaps column of Table 1–this study is positioned to make a distinct contribution to carrying capacity research in the IHR. Unlike prior assessments that emphasize ecological thresholds, spatial zoning, or tourism suitability while treating urban services only tangentially, the study explicitly centers water and sanitation–specific carrying capacity as a core analytical dimension.

**Table 1:** Summary of contribution and gaps identified through literature review in existing India specific literature

S No	Title	References	Contribution of the study	Limitation/Existing gap
1	Assessment of environmental carrying capacity of Eco-Sensitive Zone: Sanjay Gandhi National Park, Mumbai, Maharashtra	Central Pollution Control Board (CPCB), [11]	Provides a replicable, indicator-based Environmental Carrying Capacity framework using remote sensing and GIS, integrating air, water, noise, tourism, land and habitat to derive a composite ecological score	Limited to a delineated Eco Sensitive Zone; excludes urban infrastructure, service delivery systems, and dynamic population pressures, relying largely on secondary data
2	Status report carrying capacity of Mussoorie	Uttarakhand Pollution Control Board (UKPCB), [12]	Offers a rapid, diagnostic overview of geological, ecological and infrastructural stress using secondary data to flag risks from unplanned development	Lacks a defined methodology, quantitative thresholds, and integrated ecology infrastructure linkages, limiting its utility for systematic or forward-looking planning
3	Carrying capacity and strategic planning for sustainable tourism practices in the Char Dham from the Western Himalaya, India	Kuniyal et al. [15]	Uses GIS-based MCDA with multiple indicators to generate spatial suitability and capacity maps for pilgrimage destinations experiencing rapid tourism growth	Produces land-suitability zoning rather than quantifiable carrying capacity; omits sectoral thresholds, and service delivery capacities
4	Tourism carrying capacity assessment for Leh town of Ladakh region in Jammu	Shah and Sajad, [14]	Applies modified Cifuentes–Ceballos formulas to estimate tourist carrying capacity incorporating limiting factors like climate, transport, accommodation and waste management	Excludes resident population, limited to terrain and glacier-fed water dynamics, and core services such as water supply and sanitation
5	Estimating carrying capacity in a high mountainous tourist area: a destination conservation strategy	Jangra and Satya, [13]	Implements full Cifuentes PCC–RCC–ECC framework with natural and man-made correction factors for site-specific tourism management	Relies on user-satisfaction surveys instead of quantitative infrastructure metrics; neglects city-level systems, and municipal service loads

By doing so, the study responds directly to the consistent omission of municipal service delivery systems, quantifiable infrastructure thresholds, and resident–tourist service loads highlighted across existing studies. The proposed assessment framework moves beyond site-specific or diagnostic exercises by offering a pragmatic, city-level, and replicable approach that aligns water and sanitation planning with population pressures.

## Research materials and methods

**Fig. 3:**
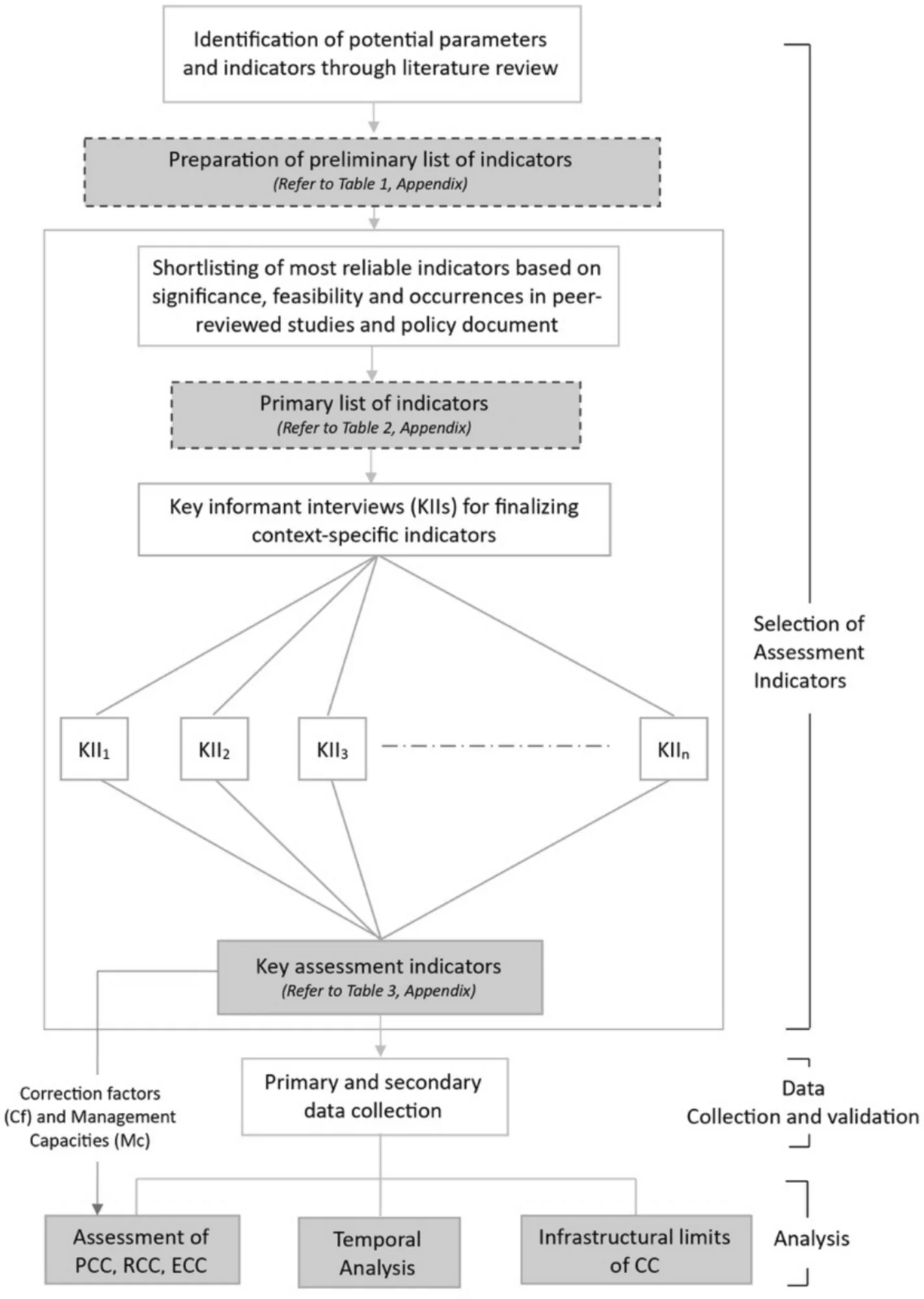
Graphical representation of the research process followed, illustrating the stepwise methodology adopted for indicator selection, data collection, and carrying capacity assessment

This study adopts a single case study design using a mixed-methods approach to comprehensively analyze carrying capacity in ecologically fragile urban areas. Given the limited existing literature, this research addresses knowledge gaps and provides new insights through expert interviews and focus group discussions. The methodology follows a structured, multi-stage process (Fig. 3), beginning with a literature review using targeted keywords (e.g., ‘carrying capacity’, ‘urban delivery’, ‘tourism’, ‘fragile regions’), forming the preliminary list of indicators (Supplementary Table 1). Furthermore, from the preliminary list of indicators reliable indicators were shortlisted based on significance, feasibility and occurrences in peer-reviewed studies and policy documents, resulting in the primary list of indicators (Supplementary Table 2). These indicators were then validated through KIIs with experts in urban planning, tourism, and sustainability (Table 2), ensuring contextual relevance to generate the key assessment indicators (Supplementary Table 3) which were employed as inputs to measure correction factors and management capacities. From the set of key assessment indicators, those directly related to climate were selected (rainfall, temperature and landslides) and subsequently utilized to conduct a temporal analysis. Data collection included both primary and secondary sources, analyzed through three components:

1. Evaluation of PCC, RCC and ECC;
2. Temporal analysis of climate and disaster-related factors;
3. Infrastructure assessment covering water, sanitation, and transport.

**Table 2:** Multi-sectoral representation of key informants interviewed for carrying capacity assessment in Nainital

Sector	Designation and department	Institution	Engagement for study	Research method	Date of engagement
Water and sanitation	Team lead, Sanitation Capacity Building Platform	National Institute of Urban Affairs	Generation of key assessment indicators	KII	02/07/2024
Urban planning	Team lead, Urban strategy unit, Sustainable Urban Development Smart Cities (SUDSC) and Master Plan Delhi- (MPD)-2041	National Institute of Urban Affairs	Generation of key assessment indicators	KII	02/07/2024
Urban planning	Sr. Urban development specialist, Sustainable Urban Development Smart Cities (SUDSC) and Master Plan Delhi-(MPD) 2041	National Institute of Urban Affairs	Generation of key assessment indicators	KII	02/07/2024
Urban planning	Dean	Ahmedabad University	Generation of key assessment indicators	KII	04/11/2024
Sustainable development	Strategist and senior researcher	–	Generation of key assessment indicators	KII	04/11/2024
City administration	Executive officer	Nainital Municipal Council	Data collection	KII	23/09/2024
City administration	Assistant engineer	Nainital Municipal Council	Data collection	FGD	25/09/2024
City administration	Mission Manager, Atal Mission for Rejuvenation and Urban Transformation	Nainital Municipal Council	Data collection	FGD	24/09/2024
City administration	Municipal Engineering (ME) department	Nainital Municipal Council	Data collection	FGD	24/09/2024
City administration	House Tax department	Nainital Municipal Council	Data collection	FGD	24/09/2024
City administration	Junior engineer	Urban Development Department, Uttarakhand	Data collection	KII	23/09/2024
City administration	Assistant engineer	Jal Sansthan, Nainital	Data collection	FGD	24/09/2024
City administration	Assistant engineer	Uttarakhand Urban State Development Agency, Project Implementation Unit, Nainital	Data collection	FGD	23/09/2024
Private agency	Head surveyor	City empaneled agency for GIS Mapping	Data collection	FGD	24/09/2024

This method ensures a rigorous, evidence-based assessment of carrying capacity for tourism sustainability in the urban context.

### Identification of Key Informant interviewees

Given the multi-sectoral nature of carrying capacity assessment, the selection of key informants was conducted to ensure a diverse representation of relevant sectors (water and sanitation, tourism, urban planning, environmental sustainability, research & academia). The identified key informants provided specialist insights into identifying the key assessment indicators that affect the carrying capacity of Nainital city.

The diverse expertise of these key informants mentioned in Table 2, facilitated a holistic evaluation of carrying capacity, ensuring that the study accounted for sectoral interdependencies and their associated policy implications. The insights provided by these informants were instrumental in refining the assessment indicators, contributing to an evidence-based framework for sustainable urban management in Nainital.

### Conduction of KIIs

KIIs and FGDs were conducted both in-person and online to ensure accessibility. Experts reviewed a preliminary list of indicators (Supplementary Table 1), developed through a literature review, and suggested additions based on their sectoral expertise. Their feedback was systematically compiled and used to finalize a contextually relevant and accurate set of key assessment indicators.

### Primary and secondary data collection

Data for this assessment were derived from both primary and secondary sources (refer to Supplementary Table 4). Secondary information was sourced from official government publications, online repositories, and archival records. These datasets were validated and updated through KIIs and FGDs, enabling triangulation across qualitative and quantitative inputs. Primary data collection involved a mixed-methods approach comprising quantitative and qualitative data-sets. A three-member research team conducted a five-day field study in Nainital city, which included site inspections, observational surveys, KIIs, and FGDs (refer to Table 2) with relevant municipal and community stakeholders.

For geospatial components, satellite-derived datasets including Digital Elevation Models (DEM) and Landsat imagery obtained from the United States Geological Survey (USGS) Earth Explorer were used to support spatial analysis. Climate variables such as temperature and rainfall were sourced from the NASA POWER database. Spatial analyses were performed using Q GIS, while climate data were processed and visualized through statistical procedures in R-Studio. The mixed methods approach employed for this study ensures data collection is multi-source (primary and secondary), triangulated, thereby robust in carrying out further assessments using the developed frameworks.

### Data analysis and evaluation of carrying capacity

The carrying capacity of the study area was assessed through three key dimensions: PCC, RCC, and ECC. Each dimension provides a distinct yet interrelated perspective on the threshold limits of visitor’s capacity. The definitions and equations of the formulas used for computing the results are presented in Table 3.

**Table 3:** Definitions and equations of formulas used for calculating the three carrying capacity limits of Nainital Municipal area

S.no	Definition	Equation (Eq)		References
1	Physical Carrying Capacity (PCC): It is the maximum number of visits that can be made to a site with a defined space, in a given time	$PCC = A \times \left( {V/A} \right) \times Rf$(Eq 1) where A is the suitable tourism area (in m^2^) V/A is the (visitors per meter) Rf (Rotation factor)		[35]
2	Visitor per meter square (V/A): The area required per tourist to do their activities comfortably. It is measured in square meters of space. The value of V/A varies depending on the activity type and size of the area	Activity/Typology	V/A	
		For moving around	1 m^2^	[35]
		Walking	2 m^2^	[37]
		Staying	5 m^2^	[37]
		Relaxation	10 m^2^	[37]
		Beach	2 m^2^	[38]
		Parks or Gardens	2 m^2^	
		Heritage sites	3 m^2^	
		Pilgrim centre	3 m^2^	
		Ecotourism site, Trek, hike	5 m^2^	
		Lake, backwater front, leisure sites	10 m^2^	
		Urban Green space per capita	9 m^2^	[39]
		Open space per capita	10–12 m^2^	[40]
3	Rotation factor (Rf): It is the number of permissible visits over a specified time calculated by dividing the total opening hours of the destination by the average time a visitor spends in the place	*Rf = open~period/average~time~of~visit*(Eq 2)		[35]
4	Real Carrying Capacity (RCC) is the maximum allowable number of visitors within a given area, once the reductive/corrective factors (Cf) derived from the particular characteristics of the site has been applied to the PCC. Real carrying capacity is also known as Ecological Carrying Capacity	$RCC = PCC \times Cf1 \times Cf2 \times Cf3 \times \ldots Cfn$(Eq 3) Where, PCC = Physical carrying capacity Cf = Correction factor or limiting factor		[41]
5	Correction factors (Cf) are restricting criteria that are obtained by considering physical, environmental, ecological, and social variables	*Cf = 1 - Ml/ Mt*(Eq 4) Where, Cf = Correction factors *Ml* = Limiting magnitude of the variable *Mt* = Total magnitude of the variable		[41]
6	Effective Carrying Capacity (ECC) is defined as the maximum number of people that an area can sustain, given the management capacity that is available at a particular place	$ECC = RCC \times Mc1 \times Mc2 \times ....Mcn$(Eq 5) Where, ECC = Effective carrying capacity Mc = Management capacity		[13]
7	Management capacities (Mc) Management capacity is determined by the facilities, infrastructure, amenities, personnel, parking, and tools that are readily available, and their number can vary with destinations	$Mc = \left( {100 - Fm} \right)/100$(Eq 6) Mc = Management capacity Fm = Management adjustment factor		[41]
8	Management adjustment factor (Fm) Represents inefficiencies, constraints, or adjustments that reduce management effectiveness	$Fm = \left( {{\mathrm{Im}} c - Amc} \right)/{\mathrm{Im}} c \times 100$(Eq 7) Imc = Ideal management capacity Amc = Real management capacity		[42]

The PCC is determined using Eq. (1) as mentioned by [35], with A, V/A, and Rf contextualized for the Nainital Municipal Area. The RCC is computed according to Eq. (3), as presented by [41]. The PCC value obtained from these calculations is subsequently multiplied by selected correction factors derived from Eq. (4) to compute the RCC, by [41]. The data utilized for the computation of these correction factors has been collected through KIIs, FGDs, field observations, and published official data sources.

The ECC has been calculated using Eq. (5), as outlined by [37, 38]. This calculation involves multiplying the RCC by the relevant management capacities. The evaluation of these management capacities is carried out using Eq. (6). The value of Management Capacity (Mc) incorporates the Management Adjustment Factor (Fm), which is derived from Eq. (7), as provided by [41]. Data concerning the Ideal management capacity (Imc) was obtained from the service delivery benchmarks and norms published by the Government of India, while the Real Management Capacity (Amc) for the selected parameters in Nainital has been obtained through key informant interviews, field observations, and published official documents, based on existing services and infrastructure following the methodology established by [41].

### Selection of Correction factors (Cf) and Management capacities (Mc) for calculating RCC and ECC

Correction factors and Management Capacities were selected from the primary list of indicators presented in the Supplementary. Both were selected from context-specific assessment indicators and supported by a literature review and KIIs with subject matter experts with prior working experience of ten years or more from sectors dealing with urban planning, water and sanitation, and sustainable development in hill terrains. Correction factors selection was based on Nainital’s ecological conditions, while management capacities reflected infrastructure availability and service delivery effectiveness. Correction factors were calculated using Eq. (4) and the management capacities were calculated using Eq. (6) and Eq. (7). The limiting magnitude of variable and the total magnitude of the variable for all correction factors were contextualized for Nainital Municipal Area. Similarly, in the case of calculating management capacities the Management adjustment factor (Fm) was derived using ideal and real infrastructure capacities of Nainital Municipal Area.

The number of correction factors and management capacities directly affects RCC and ECC values, as the equation used with each additional factor. Based on a review of four relevant, high quality studies [37, 38, 41, 43] a minimum of four and a maximum of eight correction factors were found to be typical.

For this study, four correction factors were used for RCC calculation. For ECC, four management capacities linked to a single parameter were selected. In total, ten capacities were grouped under four parameters (water, sanitation, solid waste management, tourism facilities) each represented by an average value. For instance, the water supply parameter—comprising per capita supply, lake volume, continuity of supply, and groundwater extraction—yielded an average value of 0.71. Similar averages were calculated for the other parameters and applied to RCC to derive ECC as per Eq. (5). Transportation to Nainital Municipal Area is limited to road access, and vehicle inflow during tourist season is effectively restricted to the finite number of public and private parking bays. Since transportation limits are already covered under parking management capacity, it is not assessed separately in ECC.

### Population and tourist footfall

The projected residential population of Nainital for 2025 is estimated at 45,792 using the Arithmetic Mean Method, based on census data from 2001 (38,630) [44] to 2011 (41,377) [19]. The Nainital Municipal Area has exhibited a stable resident population over the past decades (2001–2011), growing at roughly 1 per cent annually with no major economic drivers or infrastructural expansion, while the tourist population fluctuates considerably (see Table 4). The stable low annual growth rate of the municipal area makes arithmetic mean method appropriate for the population forecasting of residential population, as it is considered reliable for cities with stagnant or slow population growth.

**Table 4:** Tourism footfall as reported by the tourism department Nainital, Uttarakhand (Tourism footfall data for the 2000–2010 was not available in month-wise disaggregated format, data for 2020 April-June is zero due to Covid-19 lockdown and travel restrictions) [46]

Year	JAN	FEB	MAR	APR	MAY	JUN	JUL	AUG	SEP	OCT	NOV	DEC	Annual
2000	Month wise data not available from 2000 to 2010												2,58,536
2001													3,62,732
2002													4,16,664
2003													4,46,432
2004													4,84,410
2005													5,17,748
2006													5,62,060
2007													5,89,515
2008													6,22,539
2009													7,55,278
2010													7,93,828
2011	47,024	27,496	31,596	50,312	1,04,280	2,64,793	78,327	54,494	50,699	65,042	33,056	35,838	8,42,957
2012	50,754	29,606	33,903	54,700	1,12,667	2,85,998	84,482	58,837	54,748	67,439	35,819	37,380	9,06,333
2013	54,730	33,701	37,491	59,815	1,21,757	2,54,506	18,288	16,353	32,864	47,376	31,679	35,758	7,44,318
2014	53,002	32,913	34,324	55,119	1,12,007	2,72,641	20,700	19,606	34,504	49,370	34,542	39,395	7,58,123
2015	34,894	56,181	37,109	57,488	1,17,695	3,00,683	22,410	42,840	35,636	53,424	37,407	42,533	8,38,300
2016	53,285	37,919	39,788	62,224	1,26,992	3,82,748	24,336	12,392	37,566	56,213	39,403	44,526	9,17,392
2017	56,462	40,219	42,035	66,254	1,37,497	3,59,682	24,495	15,777	33,835	53,598	41,539	46,268	9,17,661
2018	57,078	43,416	48,098	71,303	1,48,082	3,25,950	26,126	18,172	36,017	59,676	43,457	56,286	9,33,661
2019	61,053	51,600	52,007	76,690	1,56,283	3,50,829	26,998	22,999	24,698	27,306	26,969	56,474	9,33,906
2020	60,766	52,109	39,175	–	–	–	57	118	575	15,505	16,125	31,319	2,15,749
2021	32,918	31,987	36,709	28,411	8,779	18,872	22,898	24,775	24,974	25,407	33,583	36,946	3,26,259
2022	23,538	23,946	34,850	40,201	73,120	89,561	49,840	32,589	28,809	34,097	42,215	56,909	5,29,675
2023	37,960	34,299	50,100	57,636	89,888	1,58,721	56,971	34,471	37,688	69,491	70,083	87,189	7,84,497

In line with UNWTO guidelines, this study differentiates between ‘visitors’ and ‘tourists’. A visitor is anyone traveling outside their usual environment for less than a year for purposes other than employment. Tourists are overnight visitors, while same-day travelers are also considered visitors [45].

For this study, ‘visitor’ includes both same-day and overnight guests, as well as those visiting for either leisure, medical, educational, or judicial purposes.

### Infrastructure-dependent capacity limits and temporal variability of indicators

Infrastructure-dependent carrying capacity and temporal variability were assessed using data from government sources, academic literature, and KIIs and FGDs with municipal officials involved in city planning and urban service delivery. These limits were reported in terms of ‘users’–individuals staying overnight in Nainital including residential population.

Climatic data (1990–2024) on temperature and rainfall were sourced from the NASA Power database (database with an accuracy of 95 per cent) and analysed using Microsoft® Excel to identify trends. Landslide data, covering 1867–2024, were gathered from municipal records and newspaper archives, and verified using historical maps [47, 48] to account for changes in area names.

This integrated approach facilitated the analysis of links between infrastructure, climatic variability, and carrying capacity. Pearson’s correlation coefficients were used to examine statistical relationships, with assumptions and formulas detailed in the Supplementary.

### Development of carrying capacity framework

The methodology for developing the framework for assessing carrying capacity for ULBs across IHR involved a structured, multi-step approach. First, a comprehensive Criteria Catalogue was formulated by reviewing established standards and adapting context-specific indicators relevant to urban water and sanitation systems. This was followed by designing a multi-pathway data collection strategy integrating primary surveys, secondary datasets, and climatic records. The framework then organised these inputs into three analytical pathways indicator-based evaluation, infrastructure assessment, and climate analysis to capture physical, managerial, and environmental dimensions of carrying capacity. Each pathway employed standardised calculations, benchmarks, and trend analyses to derive capacity limits and risk profiles. Finally, the insights from all pathways were synthesised into an integrated output to guide planning processes such as city master plans and tourism development.

## Results

The following section presents the results of the study for the city of Nainital, Uttarakhand.

### Calculation of PCC, RCC and ECC for Nainital Municipal Area

#### PCC

The PCC of the Nainital Municipal Area was calculated using Eq. (1). The area suitable for public use (A) was derived by subtracting the reserved forest area (A2) from the total municipal area (A1) using Eq. (8). The area of Naini lake is being extensively used for recreational purposes and is a major public use area. Hence, it is included in the area suitable for public use, with A1 as 11,730,000 m^2^ and A2 as 1,302,700 m^2^, the resulting public-use area was 10,427,300 m^2^.

8 $$A\left( {m^{2} } \right) = A1\left( {m^{2} } \right) - {A2} \left( m^{2} \right)$$

For PCC estimation, the Urban and Regional Development Plans Formulation and Implementation (URDPFI) Guidelines [40] were applied, recommending 10–12 m^2^ of open space per person in small towns. As Nainital (population 41,377) falls under the Small-Town II category, a V/A ratio of 1/12 was adopted [40].

Since, the Nainital Municipal Area has no restrictions for the entry and exit of tourists, the open period is considered to be 24 h. Taking into account the overnight tourists in Nainital, the average time of visit is also considered to be as 24 h. Hence, the rotation factor for Nainital Municipal Area is 1.

The PCC computed for Nainital Municipal Area, considering the suitable area for public use (A) as 1,04,27,300 m^2^, visitor per meter square (V/A) as 1/12 and rotation factor (Rf) as 1, is 8,34,184 persons per day.

#### RCC

For calculating the RCC of Nainital Municipal Area, correction factors classified under topography and climate are slope and rainfall, and correction factors under ecologically vulnerable sites are identified as urban green cover and landslide-prone areas, given their impacts on the city’s carrying capacity. The correction factors are explained in detail in Table 5.

**Table 5:** Correction factors identified for the calculation of real carrying capacity (RCC) of Nainital municipal area

S.No	Parameter	Correction factor	Description
1	Topography and climate	Slope	According to [49], any area having a slope above 30° is not suitable for construction and is prone to landslides
2		Rainfall	Nainital receives average annual rainfall of 1500 mm, with the highest rainfall reaching 4773 mm, leading to an increase in chances of road blockages, slope instability and landslides [25]
3	Ecologically vulnerable areas	Urban green cover	Urban green cover includes all types of visible green vegetation in the city, such as city forest, reserved forest area, agriculture land, riparian buffer along streams and Naini Lake littoral zone, recreational parks, gardens, and vegetation along streets. Urban green cover is a limiting factor as biodiversity needs to be protected from anthropogenic activities [50]
4		Landslide prone areas	Nainital is located between 1380 m and 2542 m above the mean sea level in the Outer Lesser Himalaya, to the north of the Main Boundary Thrust, which is one of the major Himalayan fault lines. More than half of the area in the city is prone to a high to very high risk of landslides [51]

The values of correction factors are calculated using Eq. (4) as mentioned in Table 3, and values for the limited magnitude of the variables (Ml) and total magnitude of the variables (Mt) required for the calculation are described in Table 6.

**Table 6:** Evaluating the value of correction factors identified for the calculation of RCC of Nainital Municipal Area

Cf (n)	Correction factor	Limiting magnitude of variable (Ml)	Data source for (Ml)	Value of Ml	Total magnitude of variable (Mt)	Value of Mt	Value of Cf using Eq. (4)
Cf 1	Slope (m^2^)	Total area having slope above 30° in Nainital Municipal Area	Slope Analysis in GIS using DEM data retrieved from the USGS Earth Explorer Data Portal	34,25,400 m^2^	Municipal Area of Nainital	1,17,30,000 m^2^	0.71
Cf 2	Rainfall days	Total number of days with moderate to extremely heavy rainfall	Number of days analyzed using rainfall data for 2024 from the NASA Power Database	21 days	Total number of days in a year	365 days	0.94
Cf 3	Urban green cover (m^2^)	Urban green cover in Nainital Municipal area	Landcover analysis for the year 2023 using landsat data retrieved from the USGS earth explorer data portal	27,44,600 m^2^	Municipal area of Nainital	1,17,30,000 m^2^	0.77
Cf 4	Landslide prone area (m^2^)	Area under moderate to high-risk zone of landslide susceptibility in Nainital Municipal area (sq m)	Landslide susceptibility map for the year 2023 prepared using the methodology outlined by [51], excluding parameters such as dissection index, ruggedness index and landform	76,24,500 m^2^	Municipal area of Nainital	1,17,30,000 m^2^	0.35

To estimate the RCC, four correction factors– slope, rainfall, urban green cover, and landslide-prone areas–were applied. The landslide-prone area factor (0.35) had the greatest impact, while rainfall (0.88), urban green cover (0.77), and slope (0.71) showed lesser influence. After adjustments to the PCC using these factors, the real carrying capacity (RCC) for Nainital was calculated as 1,49,230 persons per day, representing the maximum number that can be ecologically sustained within the municipal area.

#### ECC

For the calculation of ECC, the Management capacities (Mc) are broadly classified under the parameters of water supply, sanitation, solid waste management and Tourism facilities. The management capacities for the Nainital Municipal Area are explained in detail in Table 7.

**Table 7:** Management capacities (Mc) identified for calculation of Effective Carrying Capacity (ECC) of Nainital municipal area

S. No	Parameter	Management capacity (Mc)	Description
1	Water supply	Per capita water supply	It is the average volume of water supplied per day to every individual. It also indicates how well the city can sustain its water demand without compromising the environmental resources
2		Maximum safe volume of the lake	The volume of Naini Lake directly affects Nainital’s ability to sustain its population by influencing water availability, recreational opportunities, supporting biodiversity and environmental sustainability
3		Continuity of water supply	Continuity of water supply ensures reliable access to clean water for residents, industries, and services in the city
4		Annual rate of groundwater extraction	Groundwater is a primary source of water for Nainital. Hence, annual rate of groundwater extraction is crucial because they determine the long-term availability of water resources for residents, and industries, including tourism and agriculture
5	Sanitation	Coverage of the sewerage network	Sewerage coverage in Nainital Municipal Area is a critical factor because it directly impacts public health, environmental sustainability, and overall livability
6		Wastewater treatment	Wastewater treatment capacity determines how much wastewater the city can treat to safeguard the environment and public health
7	Solid waste management	Waste collection and recycling	Efficient solid waste collection and recycling ensure a city can sustain its population without environmental degradation, pollution, or public health risks. Proper waste management preserves urban cleanliness, sustainability, and the destination’s appeal to visitors
8		Provision of dustbins	The provision of dustbins in a city ensures proper waste management, preventing littering and environmental pollution. Adequate waste disposal infrastructure supports hygiene, public health, and the overall sustainability of urban tourism
9	Tourism facilities	Parking	Parking facilities is crucial as it reflects the ability to accommodate residents and visitors without causing congestion, inefficiency, or urban stress. Limited parking can lead to traffic jams, illegal parking, and reduced accessibility, affecting overall livability as well as tourism experience
10		Accommodation	Accommodation is a key indicator as it determines the maximum number of visitors the city can host without overcrowding. Limited accommodation can lead to rising prices, strain on infrastructure, and decreased visitor satisfaction

The value of management capacities is calculated using Eq. (6) and Eq. (7), Amc, Imc and Fm required for the calculation are described in Table 8.

**Table 8:** Evaluating the value of management capacities (Mc) identified for the calculation of ECC of Nainital municipal area

Mc (n)	Management capacity	Real management capacity (Amc)	Value of Amc	Ideal management capacity (Imc)	Value of Imc	Value of Fm using Eq. (7)	Value of Mc using Eq. (6)
Water supply							
Mc 1	Per capita supply lpcd	Per capita water supply in Nainital in liters per capita per day (lpcd)	112 lpcd^a^	Per capita water supply according to the Service level benchmark, MoHUA	135 lpcd^2^	17.04	0.83
Mc 2	Maximum safe volume of the lake (m3)	The average volume of the lake equivalent to 10 feet from the lake bed in m^3^	2,258,820 m^3 1^	The maximum safe volume of the Lake equivalent to 24 ft from the lake bed in m^3^	4,587,914 m^3 1^	50.77	0.49
Mc 3	Continuity of water supply (hrs.)	Continuity of water supply in Nainital in hrs	18.5 h ^1^	Continuity of water supply according to the Service level Benchmark, MoHUA	24 h ^2^	22.92	0.77
Mc 4	Annual rate of groundwater extraction (per cent)	Rate of annual groundwater extraction in Nainital District (2023) in per cent	52 per cent ^3^	Maximum safe rate of annual groundwater extraction in per cent according to CGWB	70 per cent ^3^	25.67	0.74
Average management capacity for water supply (McW)							0.71
Sanitation							
Mc 5	Coverage of the sewerage network	Sewerage Network Coverage in Nainital in per cent	95 per cent^1^	Sewerage Network Coverage according to Service level Benchmark, MoHUA in percent	100 per cent^2^	5	0.95
Mc 6	Wastewater Treatment	Existing wastewater treatment capacity of Nainital in MLD	2.5 MLD^1,3^	The total amount of wastewater generated in Nainital	6.12 MLD ^4^	59.15	0.41
Average management capacity for sanitation (McS)							0.68
Solid waste management							
Mc 7	Waste collection and recycling	Amount of Waste recycled in Nainital, 2024, in tons/day	5 tons/day ^1^	Amount of waste collected in Nainital, 2024 in tons/day	25 tons/day ^1^	80	0.20
Mc 8	Provisions of Dustbins	No. of dustbins provided in the Nainital Municipal Area	85 ^1^	No. of Dustbins to be provided according to the National Action Plan for Municipal Solid Waste Management, CPCB, 2015	130 ^4^	34.62	0.65
Average management capacity for solid waste management (McSW)							0.43
Tourism facilities							
Mc 9	Parking	Total no. parking spaces in Nainital (govt. owned)	685 ^5^	Total no. of cars entering Nainital in peak tourist season per day	2000 ^1^	65.75	0.34
Mc 10	Accommodation	Total no. of overnight beds in Nainital including hotels and homestays	7226 ^5^	Total no. of annual average overnight visitors in Nainital in peak tourist season per day	35,000 ^1^	79.35	0.21
Average management capacity for tourist facilities (McT)							0.27

^1^Data collected through Key Informant Interview (KII) with city officials

^2^[63]

^3^ [64]

^4^ [65]

^5^ Secondary Data collected through city officials

To estimate the ECC, ten management capacities across water supply, sanitation, solid waste management, and tourism facilities were considered. Among water supply indicators, the maximum safe volume of the lake had the highest influence (0.49). In sanitation, wastewater treatment capacity (0.41) was found to be more significant than sewerage coverage. For solid waste management, waste collection and recycling showed the lowest value (0.20). In tourism facilities, accommodation capacity (0.21) was lower than parking.

The average management capacity values for water supply, sanitation, solid waste, and tourism were calculated as 0.71, 0.68, 0.43, and 0.27, respectively. Tourism and solid waste management capacities were the lowest. These average values were applied to the RCC to derive the ECC using Eq. (5).

Based on this calculation, Nainital’s ECC was estimated at 8,422 persons per day – representing the maximum number of people the area can sustain daily, considering ecological and infrastructural limitations.

#### Calculation of PCC, RCC and ECC using different values of (V/A)

The calculation of PCC incorporates the value of visitors per meter square (V/A), for this study the value is considered to be 1/12 based on the open space required per person in URDPFI guidelines, 2014. The value 1/12 is standardized according to the guidelines but is not contextualized to the hill regions. Hence, the values of PCC, RCC and ECC are also calculated for values of (V/A) mentioned in the literature.

The values of PCC, RCC and ECC, calculated based on different values of V/A mentioned in literature are consolidated in Table 9. The results show a direct correlation between the number of visitors per square meter and the values of carrying capacities, as the highest value of visitors per square meter i.e., 1/1 yields the highest value of PCC, RCC and ECC i.e. -1,04,27,300; 18,65,373 and 1,05,272 respectively.

**Table 9:** Calculation of PCC, RCC and ECC using different values of V/A mentioned in the literature

S. No	V/A	Source	PCC	RCC	ECC
1	1/1	Cifuentes, [35]	1,04,27,300	18,65,373	1,05,272
2	1/2	Jangra & Kaushik, [36]	52,13,650	9,32,687	52,636
3	1/5	Jangra & Kaushik, [36]	20,85,460	3,73,075	21,055
4	1/9	Pouya & Aghlmand, [38]	11,47,003	2,05,191	11,580
5	1/12	Ministry of Housing and Urban Affairs [39]	8,34,184	1,49,230	8422

### Assessment of infrastructure-dependent maximum carrying capacity limits for Nainital municipal area

This study assesses Nainital’s carrying capacity by evaluating the adequacy of existing public infrastructure to support tourism while maintaining ecological balance. It estimates the maximum annual visitor load based on optimal use of key infrastructure under realistic conditions, as detailed in the Supplementary. The analysis covers five core sectors: water supply, wastewater management, public sanitation, vehicle parking, and lodging, offering an evidence-based understanding of the city’s tourism capacity.

#### Demographic context and baseline infrastructure

Based on Census projections (2001 and 2011), Nainital’s resident population is estimated to reach 45,791 by 2025 (using arithmetic mean), with an average daily tourist influx of 35,000 during peak months. The city has 24 public toilet facilities in key tourist areas, 685 designated ECS parking spaces, and 3,586 hotel and homestay rooms, reflecting substantial tourism infrastructure (KII with Municipal Council, Nainital).

#### Water supply and maximum visitor capacity

The total water abstraction for Nainital has been estimated at 9 MLD during peak tourism season and 8 MLD during non-peak season (data from Jal Sansthan, Nainital). With a current delivery of 50 lpcd to the resident population and 15 per cent Non-Revenue Water assumed (through KII), the remaining supply has been allocated to commercial and institutional use at 150 lpcd, according to service level benchmarks. Based on this, the maximum daily visitor capacity besides the resident population has been calculated at 35,736 individuals.

#### Wastewater management and maximum visitor capacity

Wastewater generation in Nainital has been estimated as 80per cent of the total water supplied. To ensure operational efficiency, 15 per cent of total STP capacity is reserved as a buffer for system variability and future needs. Four STPs have been installed–at Harinagar, Krishna Nagar, and Bheemtal – with a total capacity of 12.125 MLD [51]. Based on the Initial Environmental Examination Report, the 10 MLD Roosi STP was rendered non-functional due to past landslide damage [52] and remains non-functional (KII with Municipal Officer). Consequently, the effective treatment capacity of the three operational STPs is 2.125 MLD.

Based on an average residential water supply of 50 lpcd (through KII) and corresponding wastewater generation of 40 lpcd, residential wastewater generation has been estimated at 1.83 MLD utilizing about 86 per cent of the available treatment capacity. The remaining 14 per cent (0.295 MLD) has been allocated for commercial and institutional users. Under these conditions, the city’s treatment capacity has been found sufficient to accommodate wastewater from an additional 2,445 commercial and institutional users.

#### Parking infrastructure and maximum visitor capacity

In the assessment of parking infrastructure, based on average vehicle occupancy– four per four-wheeler and two per two-wheeler– the city’s parking infrastructure can support a maximum of 685 vehicles daily, accommodating around 2,740 visitors per day.

#### Lodging infrastructure and maximum visitor capacity

Nainital, as a major hill station, has a well-developed lodging infrastructure, including an increasing number of homestays. With 3,586 rooms and 7,226 beds across all accommodation types, the city can accommodate up to 7,226 visitors per day at full occupancy.

#### Public sanitation and maximum visitor capacity

A total of 24 public toilet facilities have been provided across key tourist areas in the city. Assuming each block contains 5 men’s and 5 women’s water closet (WC) units, the total available capacity amounts to 240 units each for men and women. Based on CPHEEO guidelines, [53] the existing infrastructure has been estimated to accommodate a maximum daily footfall of 25,200 individuals-15,600 men and 9,600 women.

**Fig. 4:**
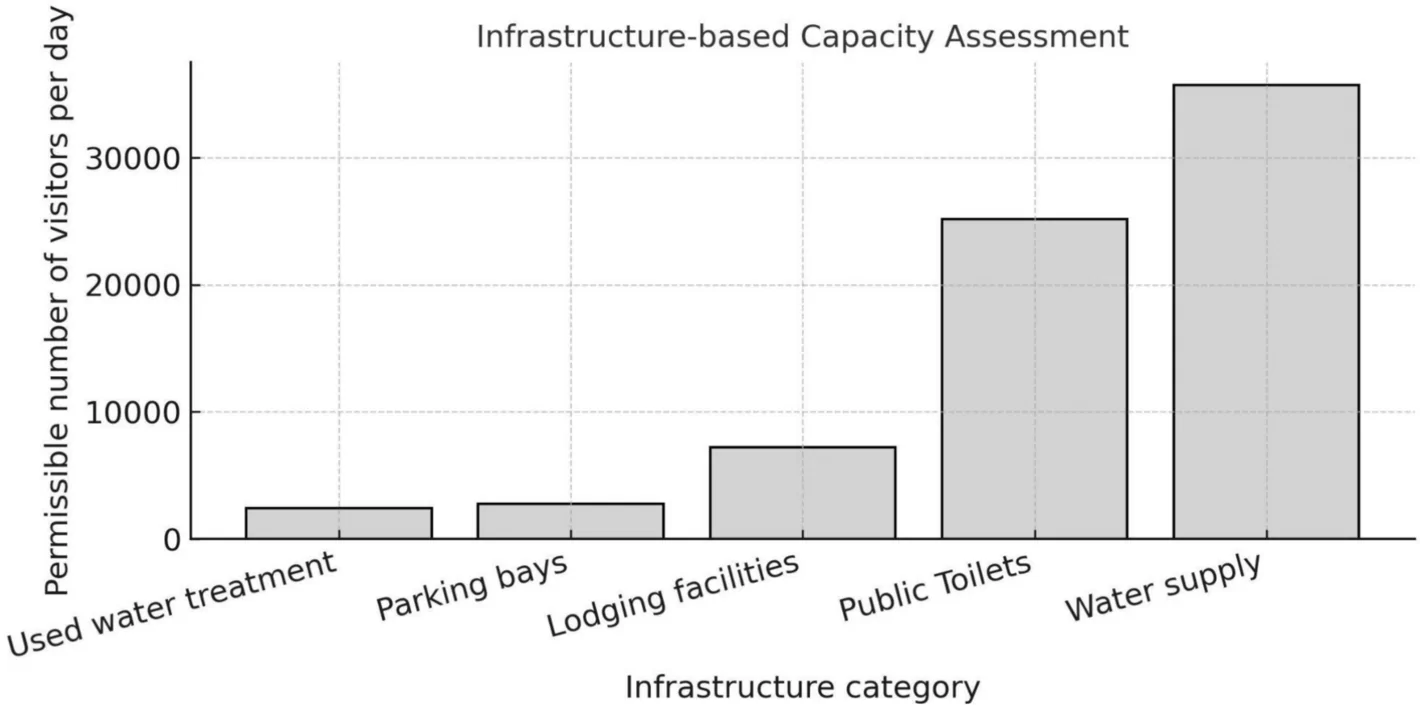
Permissible number of visitors per day based on infrastructure capacity across different categories. The graph illustrates the carrying capacity for essential infrastructure, highlighting the highest capacity for water supply and public toilets, while wastewater treatment and parking bays have the lowest permissible limits

Figure 4 presents the permissible daily visitor numbers in Nainital Municipal Area based on the carrying capacity of key infrastructure. Significant variation was observed across categories, reflecting the limitations of essential services in managing tourism sustainably.

Water supply and public toilets were found to support the highest visitor loads-35,736 and 25,200 persons per day, respectively-indicating sufficient capacity in these areas. In contrast, wastewater treatment emerged as a constraint, highlighting risks to environmental and public health. Parking and lodging capacities were estimated at 2,740 and 7,226 visitors per day, respectively, pointing to space limitations for vehicles and overnight stays.

These results emphasize the need for targeted infrastructure planning. While water and sanitation facilities are relatively adequate, limitations in wastewater treatment, accommodation, and parking must be addressed to support peak tourist inflows, which can reach up to 35,000 daily.

### Temporal development and correlational nature of climatic indicators in the primary list of indicators

This subsection presents the temporal evolution of key climatic indicators outlined in the primary criteria catalogue, namely temperature, rainfall, and disaster occurrences. Furthermore, it examines potential correlations between each criterion and tourism influx over time, offering deeper insights into the interdependencies within the study area.

**Fig. 5:**
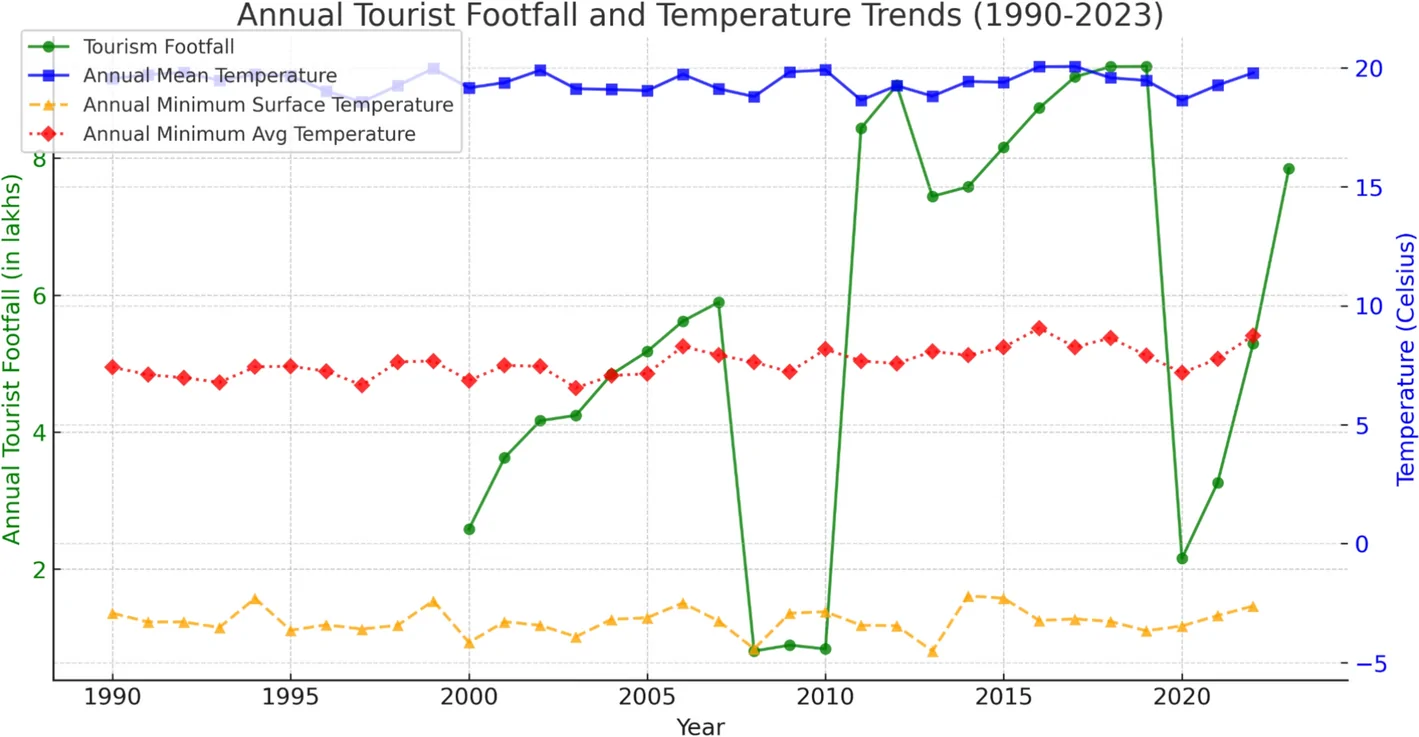
Trends in annual tourist footfall (2000–2023) and annual minimum temperature (1990–2023). The graph illustrates the relationship between annual minimum temperature (°C) and annual tourist footfall (in lakhs per year), along with linear trend lines for temperature variations. All climatic data has been accessed through the NASA Power Database

The temporal trends in annual minimum temperature and tourist footfall from 1990 to 2023 are presented in Fig. 5. The data indicates a gradual increase in annual minimum temperature over the study period, as highlighted by the linear trend line. Although there is noticeable year-to-year variability, a clear warming trend is evident in Nainital’s minimum temperatures. Between the decades 1990–1999 and 2010–2022, the city’s annual minimum temperature increased by 0.85 °C, indicating that the coldest days are becoming warmer. This change points to a significant shift in the local climate, potentially affecting the region’s overall climatic profile.

Tourist footfall in Nainital has shown significant variation, with peaks after 2000 and noticeable declines in 2008–2010 and again between 2019–2021. During the 2008–2010 period, Dehradun recorded the highest tourist inflow in Uttarakhand (0.34 million), likely due to its status as the state capital and proximity to major destinations [54]. The decline from 2019 to 2021 has been attributed to COVID-19-related travel restrictions. These findings suggest a complex relationship between temperature variations and tourism dynamics, wherein fluctuations in tourist footfall are influenced not only by climatic conditions but also by external socio-economic and policy-driven factors.

A moderate positive correlation (r = 0.54) between tourist numbers and average annual minimum temperatures in Nainital suggests that rising tourism may be contributing to climate change [55]. Anthropogenic activities such as infrastructure expansion, increased emissions, energy use, and poor waste management are likely accelerating local warming. While climatic challenges (disasters, rains) influence tourism patterns, the moderate correlation could indicate that tourism may also drive environmental change. These findings present a future scope of research into the interlinkages of tourism and climate change that emphasize on the need for sustainable tourism strategies to mitigate climate impacts and support long-term resilience.

The analysis highlights tourism’s potential impact on Nainital’s climate, with the warming of coldest days suggesting a link to anthropogenic activities. A moderate correlation (r = 0.54) between tourist influx and temperature rise indicates that tourism-related environmental changes may be increasing the city’s vulnerability to landslides.

**Fig. 6:**
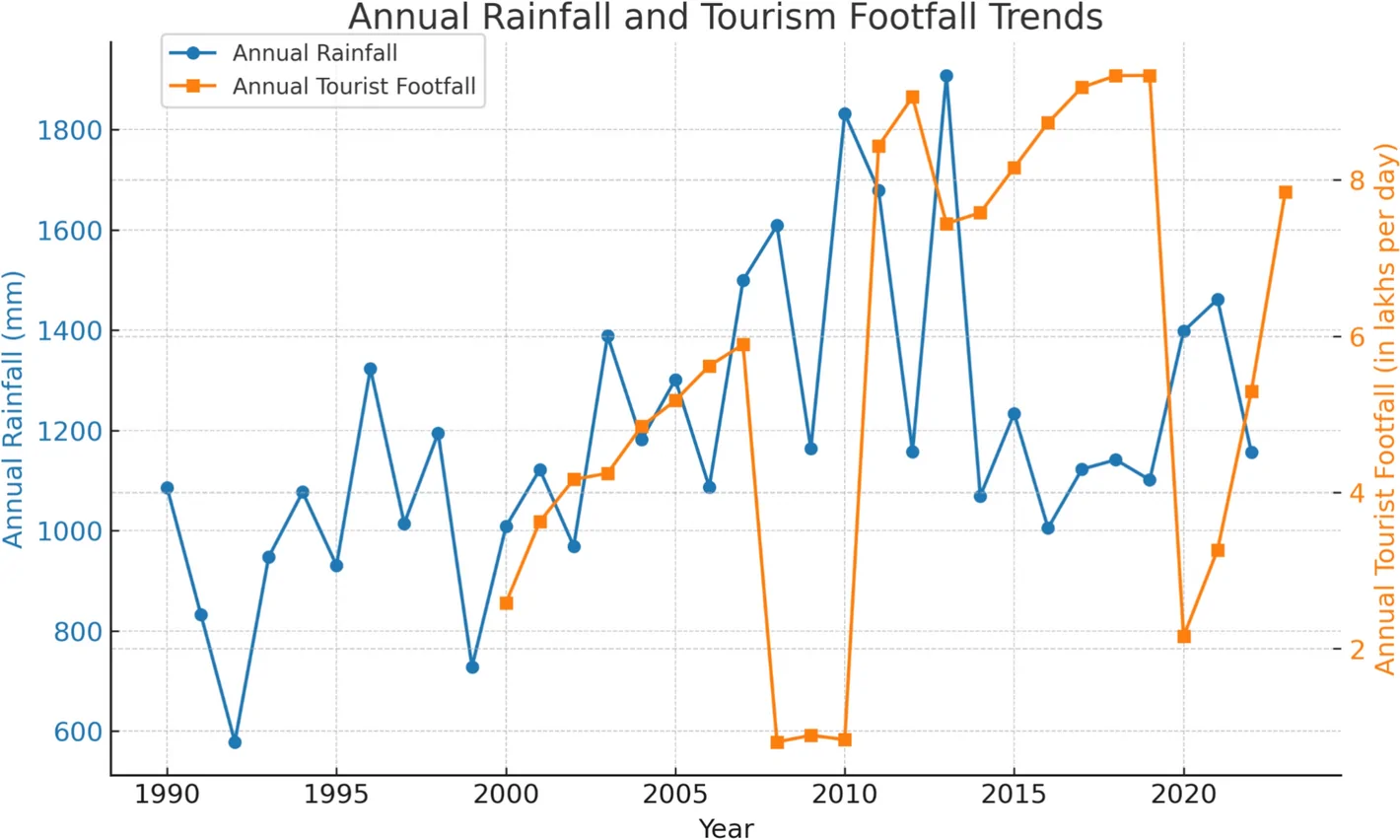
Annual rainfall and tourist footfall trends in Nainital city (1990–2023). All climatic data has been accessed through the NASA Power Database

**Fig. 7:**
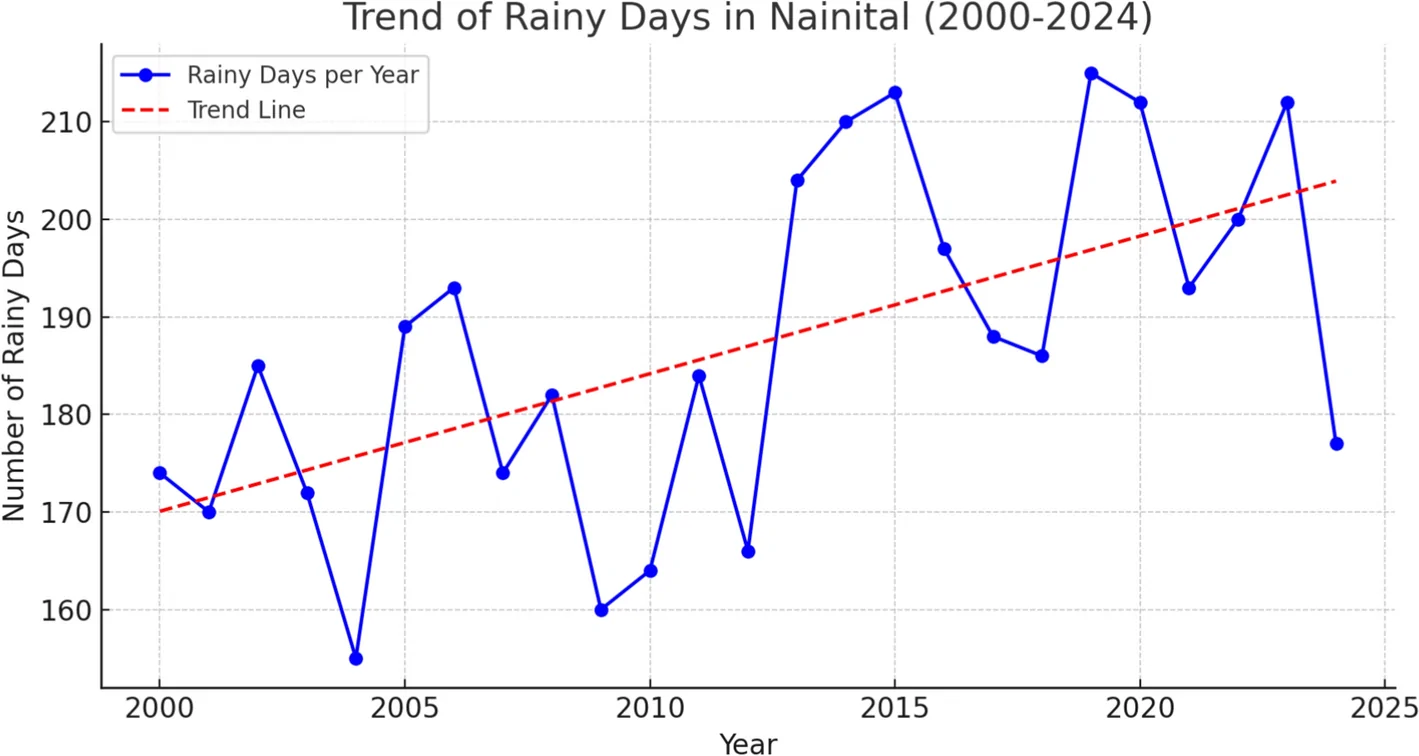
Trend in the number of rainy days in Nainital (2000–2024). All climatic data has been accessed through the NASA Power Database

Figure 6 shows the variation in annual rainfall and tourist footfall in Nainital from 1990 to 2023. Rainfall data display fluctuations, but an overall increasing trend is evident, with a 30 per cent rise between 1990–2000 and 2001–2022. Figure 7 indicates a 12 per cent rise in the average number of rainy days, from 174 (2000–2010) to 197 (2011–2024), highlighting an upward trend. These patterns suggest a potential link between increased tourism and changing rainfall trends, likely driven by infrastructure expansion, emissions, and land use changes, impacting climate and tourism dynamics.

**Fig. 8:**
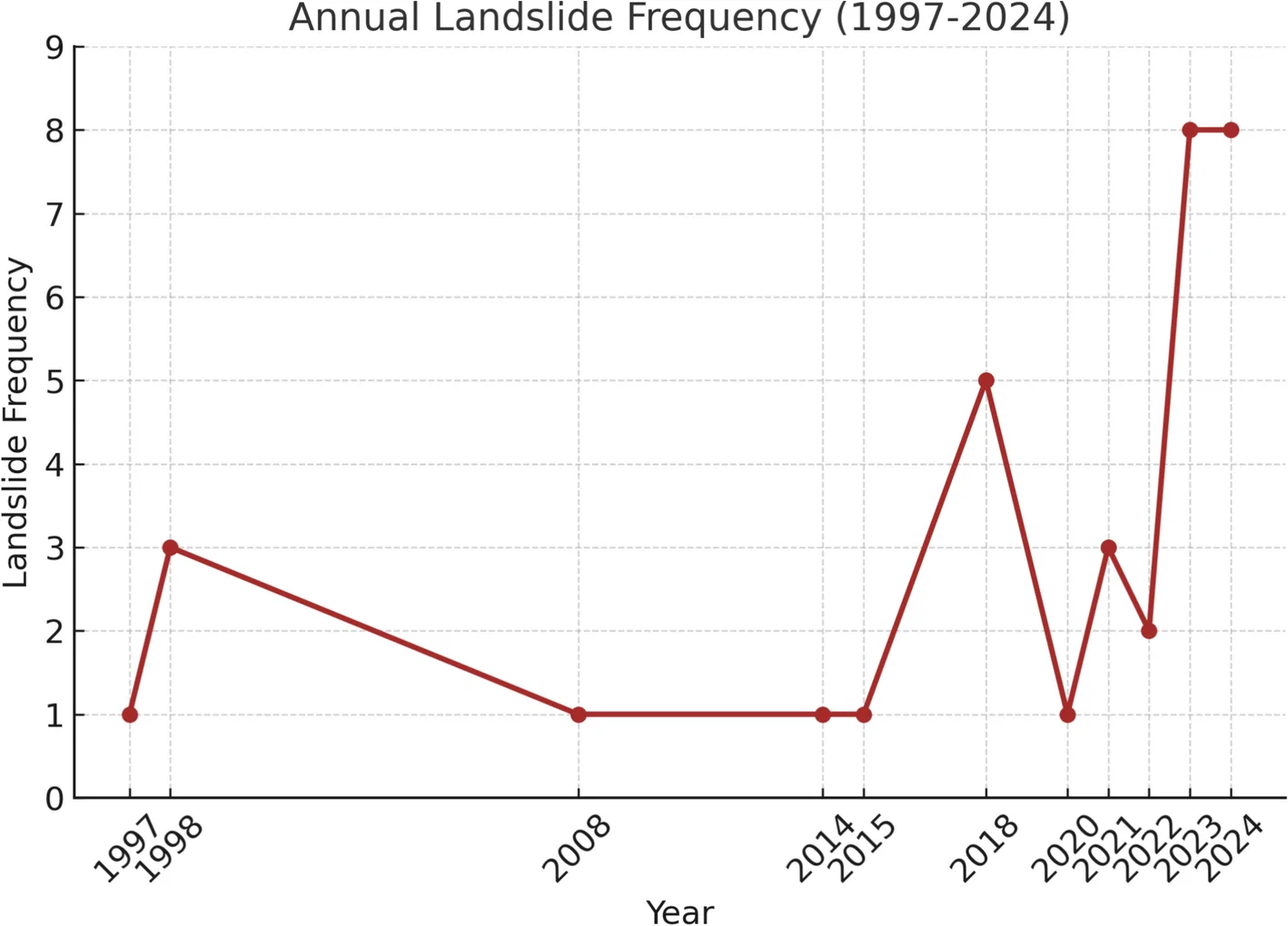
Temporal trends in landslide occurrences in Nainital (1997–2024). The graph illustrates the annual count of recorded landslide events, highlighting a significant increase in occurrences over recent years (KII and ULB records)

Figure 8 presents the temporal variation in landslide occurrences in Nainital from 1997 to 2024. The data exhibit considerable fluctuations, with relatively low occurrences between 1997 and 2015, followed by a marked increase in landslide events from 2018 onwards. A particularly sharp rise of 6 landslide counts is observed in the years 2023 and 2024, indicating an escalation in landslide frequency. The overall trend suggests an increasing vulnerability to landslides in the region over the past decade.

**Fig. 9:**
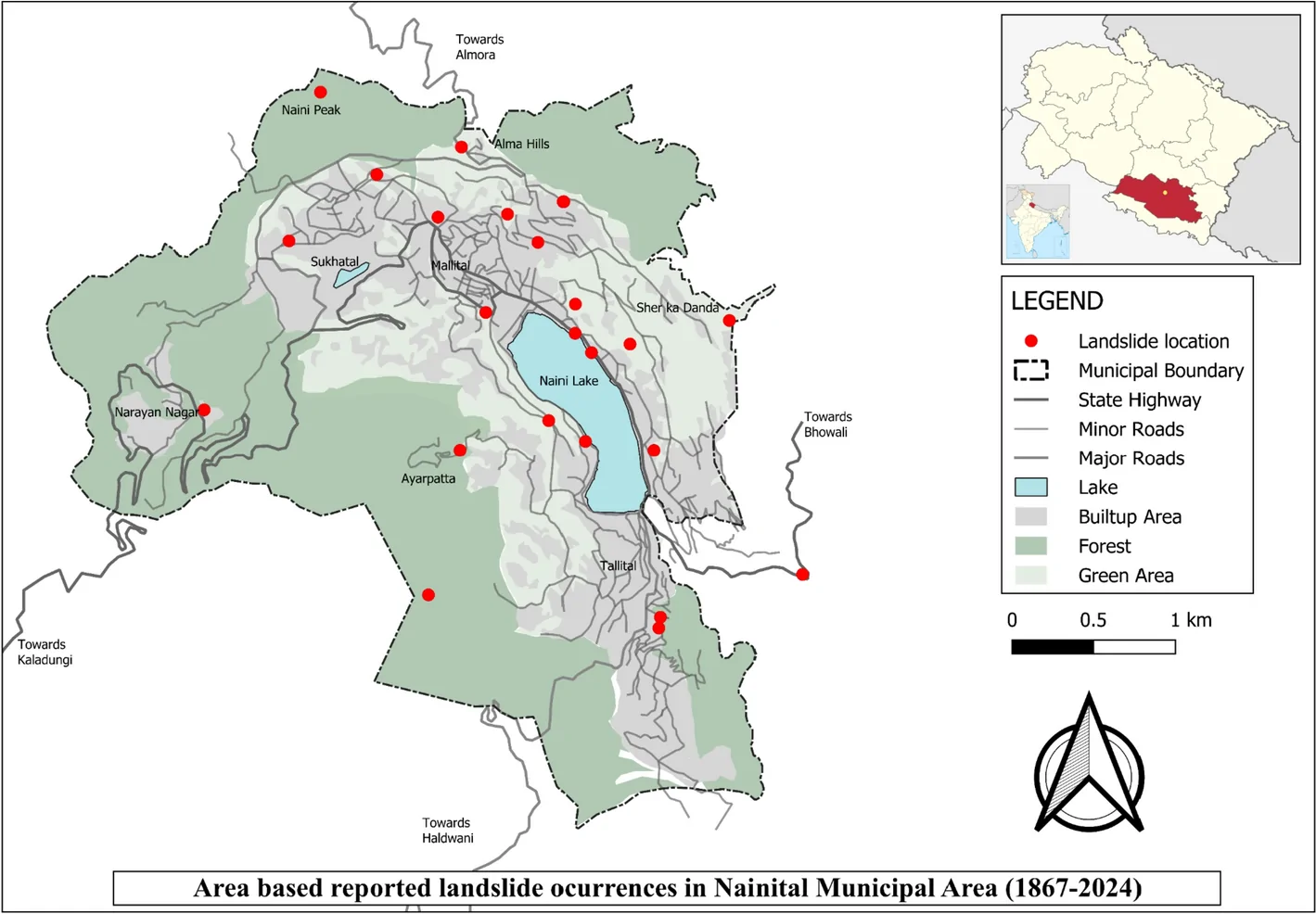
Area-based landslide occurrences in Nainital (1867–2024). The map prepared using QGIS, illustrates the area-based recorded landslide events, overlaid with the built-up area in the city [16, 56–62]

The reported occurrences of landslides within the Nainital Municipal Area from 1867 to 2024 have been illustrated as areas with at least one reported landslide occurrence, as shown in Fig. 9. This mapping underscores a significant concentration of landslide incidents in the urbanized area of the city, particularly in close proximity to Naini Lake, thereby increasing the risk to both residents and tourists. Notable locations that have experienced repeated landslides include Naini Peak, Sher Ka Danda, Mall Road, and Ayarpatta, all of which have resulted in mass movements toward Naini Lake, leading to rockfalls and the accumulation of debris within the lake. Furthermore, the Balianala landslide, which occurred in 1882 in the south-eastern part of the city, was reactivated in 2021 due to substantial rainfall, resulting in land subsidence in the area.

When analyzed in conjunction with the trends in tourism footfall and temperature variations (refer to Fig. 5: tourism & temperature), a potential correlation between increased tourism and landslide occurrences becomes evident. The period of heightened tourism activity, particularly post-2000, coincides with an increase in recorded landslide events. Tourism-induced anthropogenic activities, such as infrastructure expansion, deforestation for urban development, and increased vehicular emissions, may be contributing to the destabilization of slopes. Additionally, the rise in temperature, as previously observed, can lead to altered precipitation patterns, further exacerbating soil instability and increasing landslide susceptibility. While correlation between tourist influx, climatic parameters like rainfall, temperature, and disaster events (landslides) has been identified, further detailed research is required to be carried out to quantify and rigorously analyse tourism induced climate and disaster related events.

**Fig. 10:**
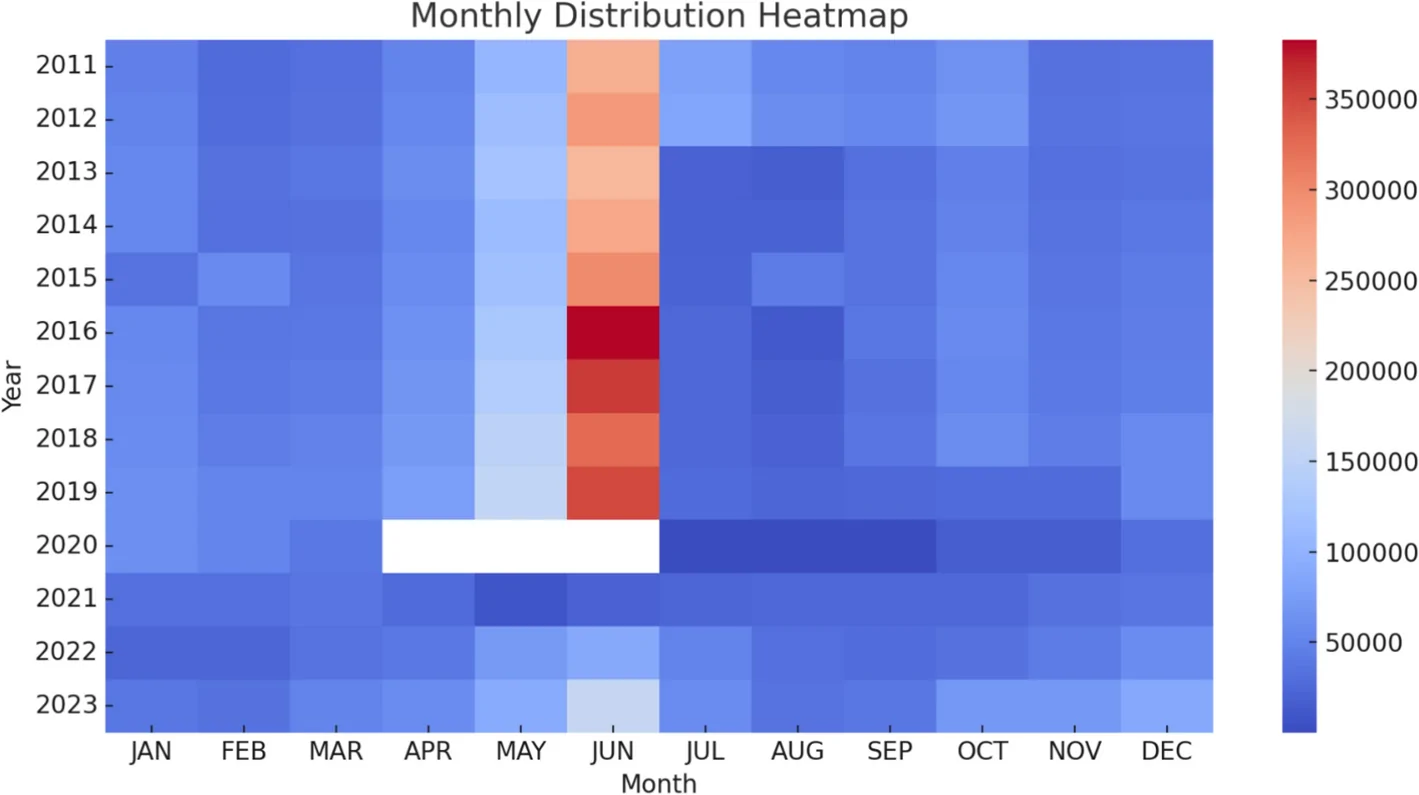
Heatmap highlighting seasonal variations and interannual trends in tourism footfall presented in monthly distribution of tourists in numbers from 2011 to 2023 based on Table 4. Color shades from blue to red indicate increasing number of tourists from 0 to 3,50,000 persons

The heatmap analysis reveals distinct seasonal trends in monthly data across the period from 2011 to 2023. As inferred from Fig. 10, May and June consistently exhibit the highest values (summer months), indicating peak activity during these months. In contrast, the period from July to September demonstrates significant declines, suggestive of monsoon months impacting tourism influx. The missing values in the year 2020 is characterized by the restrictions posed by COVID-19 disruptions that impacted activity during this period. However, a strong resurgence is evident in recent year of 2023, particularly in the later months (October to December), suggesting a recovery or shift in seasonal patterns. In recent years, the high tourism footfall seems to exist in the summer and winter months with a steady decline during monsoon months.

### Framework for carrying capacity assessment

**Fig. 11:**
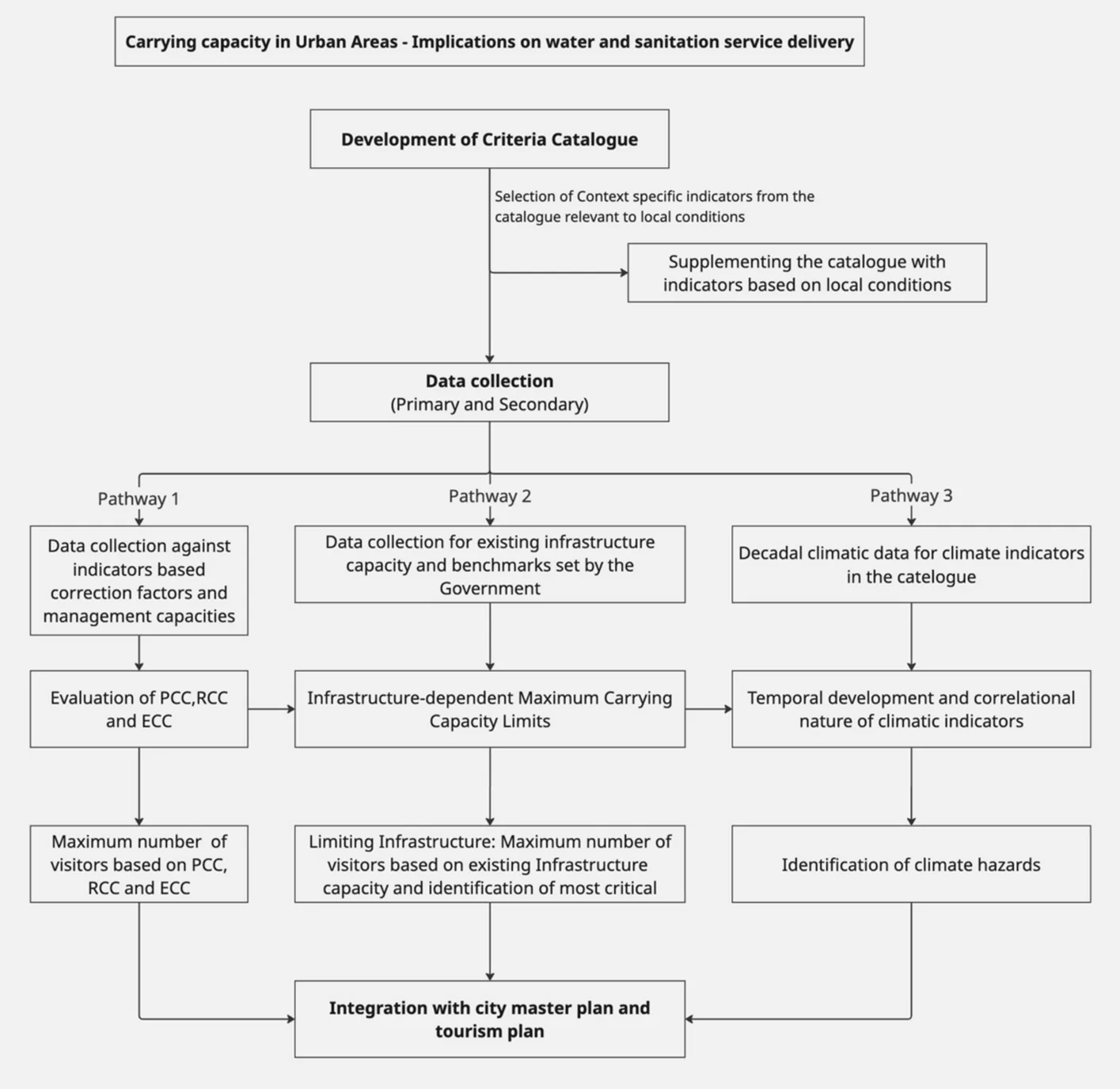
Process flowchart outlining the methodological framework for assessing urban carrying capacity and its implications for water and sanitation service delivery. The diagram illustrates the sequential steps from developing the criteria catalogue and conducting primary and secondary data collection, to evaluating physical, real, and effective carrying capacities, analyzing infrastructural limits, and integrating climatic hazard assessments to determine visitor thresholds and incorporate findings into city master and tourism planning

The flowchart (refer Fig. 11) presents a comprehensive methodological framework for assessing carrying capacity in urban areas and its implications on water and sanitation service delivery. The framework illustrates a systematic approach that begins with developing a Criteria Catalogue at the foundational level. This catalogue serves as the foundation for the entire assessment process and includes both standardized indicators and context-specific measures adapted to local conditions.

Multi-Pathway Data Collection Strategy: The framework emphasizes comprehensive data collection through both primary and secondary sources, which strategically branches into three distinct pathways, each addressing critical dimensions of urban carrying capacity.

Indicator-Based Evaluation Pathway: This pathway focuses on collecting data against indicators based on correction factors and management capacities. The collected data are subsequently evaluated for PCC, RCC, and ECC.

Infrastructure Assessment Pathway: This pathway encompasses data collection for existing infrastructure capacity and benchmarks established by government standards. These data feed into determining infrastructure-dependent maximum carrying capacity limits and identifying the most critical limiting factors. This approach recognizes that physical infrastructure often represents the most constraining factor in determining how many people or units an urban water and sanitation system can serve.

Climate Data Analysis Pathway: The third pathway incorporates decadal climatic data for climate indicators, analyzing temporal development and correlational patterns to identify potential climate hazards. This systematic analysis of climatic trends ensures that long-term climate risks are integrated into capacity assessments, addressing the growing vulnerability of urban water and sanitation services to climate variability and change.

All three analytical pathways converge toward the unified final output of integrating the findings into city master plans and tourism plan. This integrated approach ensures that urban carrying capacity assessments comprehensively account for physical infrastructure limitations, resource constraints, ecological impacts, and climate resilience factors, essential elements for sustainable water and sanitation service delivery in urban areas. The framework represents best practice methodology for assessing urban service delivery capacity in environmentally and climatically complex contexts. Its systematic integration of multiple analytical dimensions aligns well with contemporary approaches to urban water and sanitation planning in India and internationally, particularly in hill regions facing competing pressures of rapid urbanization, resource scarcity, and climate uncertainty.

## Discussion

The study’s findings highlight the critical role of carrying capacity assessments in informing sustainable planning for fragile hill cities. The numerical estimates of PCC, RCC, and ECC provide a structured analytical basis for understanding the population and tourism pressures. The reduction from a PCC of 8,34,148 persons per day to an ECC of 8,422 persons per day (refer Table 10) captures the scale of ecological and infrastructural constraints that define sustainable population thresholds in Nainital.

**Table 10:** Calculated results of carrying capacities for Nainital municipal area

Physical Carrying Capacity (PCC)	Real Carrying Capacity (RCC)	Effective Carrying Capacity (ECC)
8,34,184 persons per day	1,49,230 persons per day	8422 persons per day
**Infrastructure based carrying capacity limits**		
**Infrastructure category**	**Maximum number of users permitted (commercial and institutional)**	
Wastewater treatment	2445	
Parking bays	2740	
Lodging facilities	7226	
Public toilets	25,200	
Water supply	35,736	

The sharp difference between RCC and ECC arises from the sequential application of land-based and infrastructure-based constraints to the PCC. The RCC reflects physical and regulatory limitations on developable land. In the case of Nainital, substantial portions of the municipal area are classified as unsuitable for construction due to legally mandated and environmentally sensitive conditions. Specifically, areas designated as no-development zones under the Nainital Master Plan (2011) cover approximately 16,40,000 m^2^ (14% of the municipal area). Land with slopes exceeding 30°, as restricted under the Uttarakhand Building Construction and Development Bye-laws accounts for 34,25,400 m^2^ (29.2%), while landslide-prone areas extend over 76,24,500 m^2^ (65%). Although these categories partially overlap, together they represent a dominant share of the city’s terrain that is ideally unsuitable for built-up development.

**Fig. 12:**
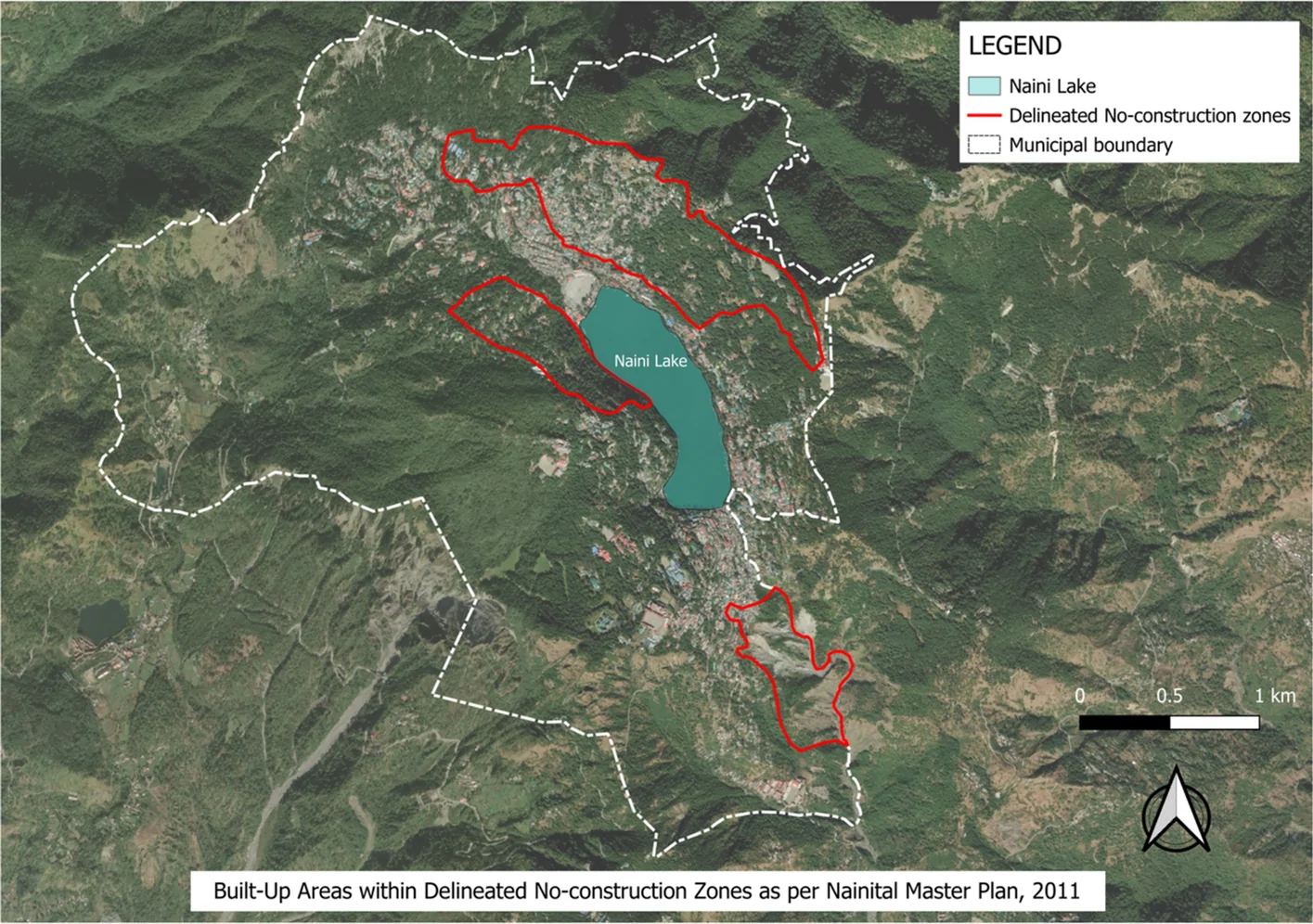
Encroachment into delineated no-construction zones (2026) as defined under the Nainital master plan (2011). Base map sourced from ESRI world imagery (January 2026), prepared using QGIS

Field observations and spatial analysis (Fig. 12) indicate that significant built-up development has nevertheless occurred within these restricted zones, including on steep slopes, landslide-prone areas, and along natural drainage channels. This reflects historical and ongoing pressures arising from population growth and tourism-driven influxes, rather than compliance with planning norms. However, for the purpose of estimating RCC, these constraints were applied under an idealised planning approach, in which slope, landslide susceptibility, and green areas, are treated as limiting factors. When these correction factors are applied to PCC, the resulting RCC is substantially reduced, explaining the sharp decline at this stage.

The further reduction from RCC to ECC is attributable to the inclusion of management and infrastructure constraints. ECC incorporates the functional capacity of existing urban systems, including water supply, solid waste management, and tourism-related accommodation infrastructure. In Nainital, these systems operate with limited capacity relative to current demand. The management capacity values derived for waste collection and recycling (0.20) and accommodation infrastructure (0.21) indicate significant service deficits. When these infrastructure-based correction factors are applied to the RCC, the ECC decreases further.

Thus, the pronounced contrast between RCC and ECC is explained by the cumulative effect of (i) stringent land-based constraints reflecting environmental sensitivity and planning regulations, and (ii) limited management and service capacities of existing urban infrastructure. This sequential filtering highlights the extent to which current settlement patterns and service provisioning in Nainital exceed beyond legal mandates, ecological suitability and institutional capacity.

These findings align with existing carrying capacity literature, which emphasises that sustainable thresholds are achieved when ecological, climatic, and service delivery factors are considered and applied to the land or area-based limits. By quantifying these limits, the study underscores the need for context-specific carrying capacity frameworks in ecologically sensitive hill cities, highlighting that uniform standards, such as the URDPFI guidelines for open space per person, are inadequate for hilly areas and fail to account for local environmental and socio-economic complexities. The reduction in ECC also necessitates targeted urban management decisions, compelling authorities to prioritise service delivery efficiency, regulate peak-season inflows, and channel investments toward the city’s most ecologically limiting sectors, particularly wastewater treatment.

Infrastructure-based carrying capacities further sharpen these implications. While the water supply system could support an additional 35,736 users per day, wastewater treatment capacity could accommodate only 2,445 more users (refer Table 10) beyond the resident population of 45,791. Water and wastewater must therefore be treated as a single, interconnected parameter in infrastructure planning. Integrating both supply and wastewater management allows resource availability, treatment capacity, and environmental load to be assessed holistically rather than in silos.

This stark imbalance positions wastewater management as the primary limiting factor for sustainable growth, with the degradation of the Nihal River illustrating the consequences of inadequate treatment capacity. It underscores that effective urban management depends not only on physical infrastructure but also on robust governance, operational efficiency, institutional capability, policy enforcement, and coordinated resource management. Consequently, policy interventions aimed at increasing the city’s ECC should prioritise the expansion of terrain-specific decentralised wastewater treatment facilities and ecological safeguards, focusing on management efficiency. Aligning infrastructure development with ecological thresholds offers a sustainable pathway to safely enhance the city’s carrying capacity.

**Fig. 13:**
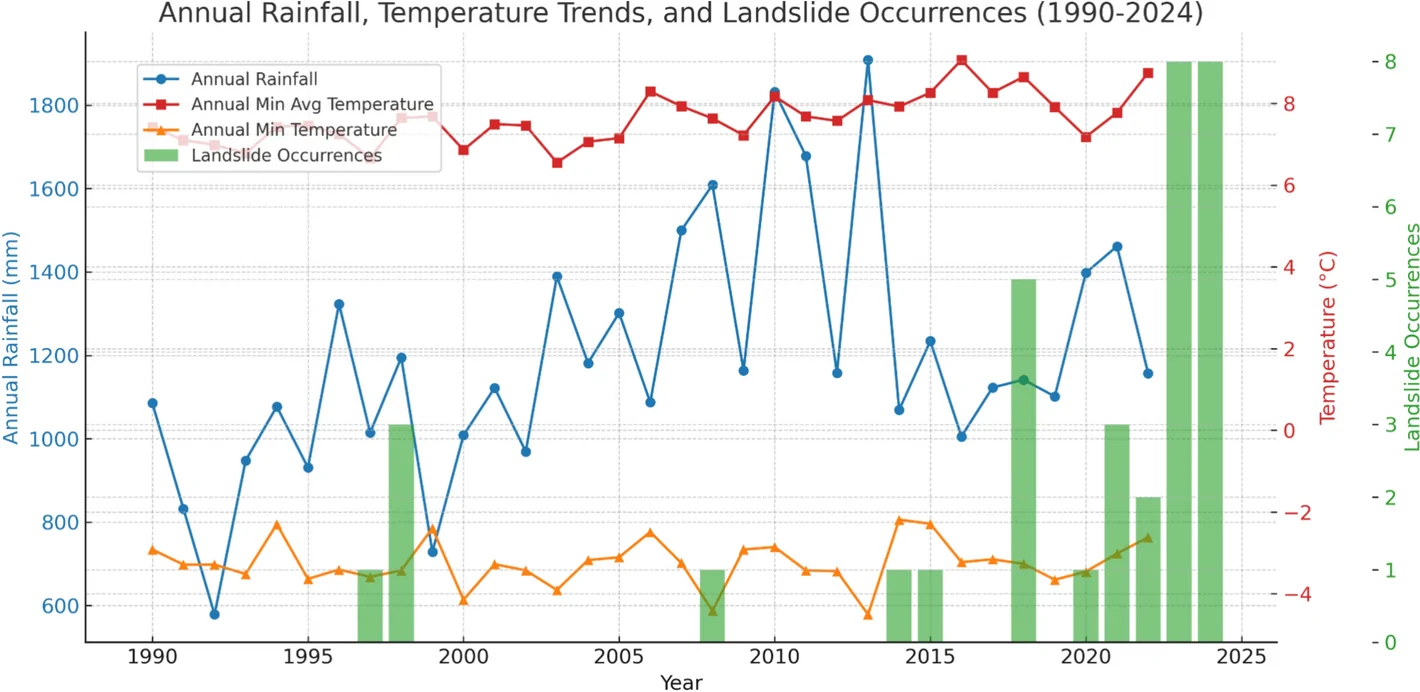
Trends in annual maximum and average surface temperatures, annual rainfall volume, and landslide occurrences over time. The temperature and rainfall trends are represented by line graphs, while landslide occurrences are depicted using bars

Figure 13 highlights the interlinkage between landslide occurrences and climatic parameters. Observed increases of 0.85 °C in minimum temperatures, 0.09 °C in overall temperature, a 30 per cent rise in rainfall, 12 per cent rise in rainy days, and a sevenfold growth in landslide incidents have influenced tourist behaviour, with a 41 per cent decline (refer Fig. 10) in monsoon arrivals and higher seasonal concentration during summer and winter. These patterns illustrate that climate variability is an active determinant of tourism flows and economic stability in hill cities. Tourism exceeding carrying capacity may amplify environmental stress, as unregulated activities like deforestation, groundwater over-extraction, and unplanned expansion intensify hazards such as landslides, water scarcity, and wastewater mismanagement. This creates a feedback loop where worsening climate conditions reduce the city’s appeal, threatening long-term tourism revenue and local economic stability.

Given tourism’s central role in Nainital’s economy, the carrying capacity framework is designed not to restrict tourism but to enable balanced, sustainable planning, while maintaining economic vitality. The quantitative thresholds provide a basis for calibrating visitor inflows, improving service delivery, and prioritising infrastructure interventions, particularly wastewater treatment and water supply enhancements, to safely increase ECC without compromising ecological integrity. The findings underscore the need for policy interventions that align sustainable tourism with climate resilience, including hazard-informed planning incorporating landslide-risk and flood-risk zoning in infrastructure development and tourism activities.

Effective planning ensures that the framework guides sustainable tourism management rather than deterring it. Alongside infrastructure and service delivery, spatial planning also emerges as critical. High court restrictions on new construction and NGT-mandated environmental zoning prevent accommodating projected tourism pressures within the current municipal area. Peak daily footfalls of 1,50,000 visitors on occasions like New Year’s Eve starkly exceed ECC, highlighting the need for strategic and sustainable regional-level tourism planning. Promoting nearby areas such as Bhimtal, Sattal, Pangot, Naukuchiyatal, Khurpatal, and Kilbury as counter-magnets allows redistribution of tourist pressure, alleviates stress on the municipal core, and aligns development with ecological carrying capacities at the regional scale. However, tourism-related development in these counter-magnets must be systematic and guided by their respective carrying capacities to ensure sustainability and avoid overburdening surrounding ecosystems.

Overall, the study demonstrates that numerical carrying capacity estimates, when integrated with climatic, socio-economic and governance considerations, provide a robust framework for sustainable urban planning in hill cities. By embedding ECC-informed thresholds into planning, infrastructure investment, tourism management, and regional development strategies, fragile hill ecosystems like Nainital can enhance environmental resilience, protect essential services, and sustain their tourism-driven economies in the face of climate and anthropogenic pressures.

## Conclusion

The study developed and applied a systematic framework for assessing the carrying capacity of ecologically fragile Himalayan hill cities, demonstrated using Nainital Municipal Area as a representative case. Acknowledging the absence of a standardized national framework, this study presents a framework that quantifies carrying capacity at a city-level and further enables ULBs in the IHR to assess and respond to NGT’s mandate for carrying capacity–based planning in hill settlements.

The analysis reveals that the combined peak daily population in Nainital exceeds the ECC value by more than 9.6 times (~ 80,791 persons/day). This substantial overshoot reinforces the urgency of implementing visitor-load regulation and upgrading critical infrastructure. However, the ECC value can be increased by strategically improving urban services and robust infrastructure. Measures such as reducing non-revenue water (NRW), increasing reliability of the water supply system, expanding sewerage networks in geotechnically sensitive areas, and investing in catchment-based decentralized STPs would directly strengthen water and sanitation performance. Complementary interventions-such as multi-level parking facilities and improved lodging infrastructure-may also enable a controlled increase in ECC while protecting environmental safeguards.

Climate and disaster related parameters show a rise in overall intensity and frequency through the decades as analyzed for Nainital Municipal Area. However, further research is required to understand complex interactions between climate change, tourism pressures, and service-delivery thresholds in mountain environments.

The proposed framework offers a transferable, evidence-based tool tailored to hill cities that integrates terrain sensitivity, climate risks, and infrastructure performance. Through integration into (a) existing city master plans and (b) the revision or formulation of state-level tourism plans, the proposed framework offers critical decision-support insights to guide sustainable planning for cities.

By identifying priority stress points-particularly wastewater treatment capacity and mobility constraints-this study supplements the design of climate-responsive tourism policies aligned with Uttarakhand’s ongoing eco-tourism initiatives aimed at decentralizing tourist activity. Moreover, operationalizing this carrying capacity framework will enable state and local authorities to drive informed tourism policies and targeted investment planning in hill cities, ensuring that future growth remains within sustainable ecological limits while strengthening economic resilience. The work demonstrates the feasibility and strategic value of integrated carrying capacity assessments for Himalayan cities, providing a pathway to reconcile ecological resilience with socioeconomic vitality under escalating tourism and climatic pressures. Therefore, this research fills a critical methodological and policy-relevant gap, providing urban local bodies and district administrations with an operational tool to estimate, evaluate, and plan for carrying capacity in high-footfall hill cities, while advancing resilient and sustainable service delivery outcomes within the IHR.

## Limitations

1. This study did not encompass the entire spectrum of tourism-induced pressures. The assessment was carried out with a primary focus on service delivery driven by water supply, sanitation, and tourism infrastructure parameters such as lodging facilities and parking bays.
2. While green cover was assessed, the study did not examine biodiversity, fauna, or broader ecological interactions in depth, given its primarily urban-centric and infrastructure focussed.
3. Transportation to Nainital has predominantly been limited to road-based, and during peak tourism periods when carrying capacity is most stressed the effective transportation capacity is naturally limited by the total number of available parking bays, both public and private. Therefore, for the case of Nainital only the aspect of road-based transport was covered under transportation.
4. Housing could not be included as an explicit carrying-capacity dimension due to the absence of disaggregated, plot- or household-level occupancy data in Nainital, which, combined with organically evolved and heterogeneous settlement patterns and the lack of enforceable residential density standards in Indian planning frameworks, prevented reliable estimation of per capita residential space.
5. Population growth was estimated using the arithmetic increase method as the Nainital Municipal Area has exhibited a stable resident population over the past decades (2001–2011), growing at roughly 1per cent annually with no major economic drivers or infrastructural expansion, while the tourist population fluctuates considerably.
6. Socio-economic indicators were included selectively based on context and not comprehensively, limiting a full understanding of social–ecological linkages.
7. The analysis was restricted to Nainital’s municipal boundary, and indicators/parameters were tailored to its specific hill context; therefore, results may not be directly transferable to other hill towns.
8. PCC, RCC, and ECC estimates are indicative and should be interpreted collectively, as assumptions, data gaps, and simplifications introduce uncertainties that may affect their validity.
9. Seasonal fluctuations in water demand, waste generation, and tourism activity were not factored into correction factors, potentially affecting the accuracy of RCC and ECC values.
10. Climatic data were included to suggest potential tourism–climate linkages; however, no rigorous statistical modelling (regression analysis, p-value, uncertainties) was undertaken, marking a potential area for future research.
11. Assumptions in correction factors, data limitations, and the exclusion of ecological and demographic variability may influence the generalisability of the findings.

## Future scope of work

Future research can build on this study by progressively advancing toward more dynamic and spatially explicit modelling approaches that better reflect the evolving nature of hill city systems. Integrating hydro-meteorological forecasts, slope stability assessments, and watershed resilience parameters would strengthen the ability to anticipate hazard-driven constraints, while incorporating community perceptions and seasonal variations in tourism and service demand would enhance the relevance of the analysis for local decision-making.

Drawing from the findings of this assessment, subsequent work should also explore the interlinkages between climate variability and tourism-related activities, including their positive and negative feedbacks on urban services. Comparative studies across multiple hill cities would enable the identification of shared and divergent stressors, support cross-learning, and help refine context-specific carrying capacity thresholds, thereby facilitating the scaling of the framework across diverse Himalayan urban contexts.

The generalisability of the findings is influenced by methodological limitations, including the absence of regression analysis, p-value estimation, and uncertainty quantification for climatic and disaster parameters, as well as assumptions embedded in correction factors, data constraints, and the exclusion of ecological and demographic variability. Addressing these gaps in future research will be critical to strengthening analytical robustness, improving model reliability, and enhancing confidence in the framework’s application for planning and policy.

For the framework to be effective at scale, its indicators must align with the varied governance structures of hill cities and be carefully contextualised to local realities. This contextualisation should be undertaken through structured engagement with subject-matter experts, local communities, stakeholders, and decision-makers, who can collectively identify relevant ecological and infrastructural constraints. Effective on-ground implementation of the framework’s findings requires engagement from urban local governments, supported by political will and ensured through strict enforcement to manage escalating tourist and resident numbers in order to maintain a positive tourism-ecology balance. Regular, preferably annual, updates of the carrying capacity assessment will be essential to ensure continued relevance and accuracy. Ultimately, multi-city applications across the Himalayan region will be pivotal in establishing benchmarks and informing the evolution of a standardized national framework for sustainable planning in mountain urban systems.

## Supplementary Information

Additional file1 (ZIP 54274 KB)

## Data Availability

Additional data supplementary to the submitted manuscript, can be accessed via this link- https://drive.google.com/drive/folders/1NzeMVVzIwHeGbx92DZVe7YFWLOkO_ofo?usp=sharing

## References

[1] Kouame A. Gearing up for India’s Rapid Urban Transformation. Economic times [Internet]. 2024 Jan; https://www.worldbank.org/en/news/opinion/2024/01/30/gearing-up-for-india-s-rapid-urban-transformation.print.

[2] IIT Guwahati, IISc Bengaluru. Climate vulnearbility assessment for the Indian Himalayan Region using a common Framework [Internet]. 2019. https://dst.gov.in/sites/default/files/NMSHE_Mission_document.pdf. Accessed 10 Dec 2025.

[3] Parida B, Arvind C, Behera M, Kumar N. Handbook of Himalayan Ecosystems and Sustainability [Internet]. 1st ed. Parida B, Arvind C, Behera M, Kumar N, editors. Taylor & Francis Group; 2023 [cited 2025 Dec 10]. 10.1201/9781003268383.

[4] Ashok M, Advisor J, Aayog N, Sejal M, Wwf W, Ms I, et al. Report of working group II sustainable tourism in the Indian Himalayan region [Internet]. 2018. https://library.niti.gov.in/cgi-bin/koha/opac-retrieve-file.pl?id=590800ca82413427e3ba16f1447e9a54. Accessed 2 May 2025.

[5] Padhi S, Pathria A, Khan Khongthaw L, Matto M, Boargaonkar R, Patil D, et al. Title rapid assessment of water and sanitation in Hill States Bremen overseas research and development Association-South Asia (BORDA-SA) Graphic Design [Internet]. 2023. https://niua.in/intranet/sites/default/files/2929.pdf. Accessed 2 May 2025.

[6] Centre for science and environment. State of India’s environment 2024 [Internet]. 2024. https://csestore.cse.org.in/default/books/state-of-india-s-environment-2024.html. Accessed 9 Dec 2025.

[7] IBEF. Uttarakhand State Report. 2024 [cited 2025 Apr 15]; https://www.ibef.org/states/uttarakhand-presentation. Accessed 15 Apr 2025.

[8] Kaur G. Cloudbursts, landslides, and floods: Why Uttarakhand’s fragile Himalayas are breaking under pressure. India Water Portal . 2025.

[9] Agrawal S, Chauhan A. A decade after the floods, is Kedarnath safer? Dialogue earth. 2023.

[10] NGT. Bird’s eye view of NGT performance in the last five years [Internet]. New Delhi; 2023. https://www.greentribunal.gov.in/sites/default/files/important_orders/NGT_Initiatives%20final-1.pdf. Accessed 10 Dec 2025.

[11] CPCB. Assessment of environmental carrying capacity of eco-sensitive zone: Sanjay Gandhi National Park, Mumbai, Maharashtra submitted in the matter central pollution control board [Internet]. 2021. https://www.greentribunal.gov.in/sites/default/files/news_updates/Report by CPCB in O.A No. 462 of 2018 (D.V Girish Vs. Union of India & Ors.).pdf. Accessed 10 Dec 2025.

[12] UKPCB. Status report carrying capacity of mussoorie [Internet]. 2023 Jul. https://www.greentribunal.gov.in/sites/default/files/news_updates/REPORT%20BY%20UK%20SPCB%20IN%20OA%20NO.%2051%20of%202023%20In%20re%20%20News%20item%20published%20in%20The%20Tribune%20dated%2016.01.2023%20titled%20Joshimath%20disaster%20a%20warning%20for%20Mussoorie.pdf. Accessed 10 Dec 2025.

[13] Jangra R, Kaushik SP. Estimating carrying capacity in a high mountainous tourist area: a destination conservation strategy. 2021. pp 427–47. 10.1007/978-981-15-4956-4_22.

[14] Dar S, Shah S, Wani M. Tourism carrying capacity assessment for Leh town of Ladakh region in Jammu and Kashmir. Int J Curr Res. 2016;2:26403–10.

[15] Kuniyal JC, Maiti P, Kanwar N, Dhyani R, Nand M. Carrying capacity and strategic planning for sustainable tourism practices in the Char Dham from the Western Himalaya, India. Sci Rep. 2025. 10.1038/s41598-025-20166-8. PMC12534453 41107367

[16] Sushil K. Natural Hazards in the township of Nainital, Uttarakhand in India. Int J Eng Appl Sci Technol. 2019;2019(3):42–9.

[17] Lee E, Carrivick JL, Quincey DJ, Cook SJ, James WHM, Brown LE. Accelerated mass loss of Himalayan glaciers since the little ice age. Sci Rep. 2021;11:24284. 10.1038/s41598-021-03805-8. 34931039 PMC8688493

[18] Tiwari PC. Land-use changes in Himalaya and their impact on the plains ecosystem: need for sustainable land use. Land Use Policy. 2000. 10.1016/S0264-8377(00)00002-8.

[19] Census of India. District census handbook, Nainital. 2011.

[20] Jha R. Lessons from Joshimath: The need for a Himalayan development model [Internet]. 2023. https://www.orfonline.org/public/uploads/posts/pdf/20240608201622.pdf. Accessed 2 May 2025.

[21] Chopra R. Joshimath: An avoidable disaster. The India forum [Internet]. 2025; https://www.theindiaforum.in/environment/joshimath-avoidable-disaster.

[22] The Hindu Bureau. 900 members of 29 families displaced in Joshimath land subsidence, says state disaster management secretary. The Hindu [Internet]. Uttarakhand ; 2023 [cited 2025 Dec 9]; https://www.thehindu.com/news/national/other-states/900-members-of-29-families-displaced-in-joshimath-land-subsidence-says-state-disaster-management-secretary/article66413601.ece/amp/. Accessed 9 Dec 2025.

[23] Kumar V, Chopra AK, Srivastava S, Maheshwari RK. Uttarakhand: Most disaster prone state of India, need for effective management [Internet]. The Science Observer. 2013. www.thehindu.com.

[24] Pande S, Bhardwaj M. Changing tourism trends and vulnerability assessment of built environment in hill stations of Indian Himalayan Region. Curr World Environ. 2024;19(1):237–50. 10.12944/cwe.19.1.21.

[25] Gupta V, Bhasin RK, Kaynia AM, Tandon RS, Venkateshwarlu B. Landslide hazard in the Nainital township, Kumaun Himalaya, India: the case of September 2014 Balia Nala landslide. Nat Hazards. 2016;80:863–77. 10.1007/s11069-015-2002-5.

[26] Divyanjali, Thakur G, Priyanka, Singh R, Gehlot A, Twala B, et al. Assessment of environmental degradation of lakes of Nainital district: an ecohydrological perspective. SN Appl Sci. Springer Nature;Singapore; 2023;5. 10.1007/s42452-023-05491-9.

[27] Dash RR, Mehrotra I, Kumar P, Grischek T. Lake bank filtration at Nainital, India: water-quality evaluation. Hydrogeol J. 2008;16:1089–99. 10.1007/s10040-008-0295-0.

[28] Dalakoti H, Mishra S, Chaudhary M, Singal SK. Appraisal of water quality in the lakes of Nainital District through numerical indices and multivariate statistics, India. Int J River Basin Manag. 2018;16:219–29. 10.1080/15715124.2017.1394316.

[29] Chauhan D, Thiyaharajan M, Pandey A, Singh N, Singh V, Sen S, et al. Climate change water vulnerability and adaptation mechanism in constrained touristic Himalayan city, Nainital [Internet]. 2021. 10.21203/rs.3.rs-571054/v1. 34331647

[30] Ministry of tourism. Status OF 48 projects under prasad scheme [Internet]. New Delhi; 2025 [cited 2025 Dec 10]. https://tourism.gov.in/sites/default/files/2025-03/usq.3716 for 24.03.2025.pdf. Accessed 10 Dec 2025.

[31] Uttarakhand tourism development board. Invest in Uttarakhand [Internet]. 2023. https://investuttarakhand.uk.gov.in/themes/backend/uploads/focus_Sector_Tourism.pdf. Accessed 9 Apr 2025.

[32] McIntyre George, Arlene Hetherington, Edward Inskeep. Sustainable tourism development : guide for local planners. Sustainable tourism development : guide for local planners [Internet]. 1993 [cited 2025 May 2]; https://search.worldcat.org/en/title/29435314. Accessed 2 May 2025.

[33] Leung Y-F, Spenceley A, Hvenegaard G, Buckley R. Tourism and visitor management in protected areas [Internet]. 2018. www.iucn.org/pa_guidelines.

[34] Mathieson A, Wall G, Reilly Merton RT, John McDonald M. Various paging. 8.50 pounds. Institute of certified Travel Agents. 1982.

[35] Cifuentes M. Determination of tourist carrying capacity in protected areas [Internet]. Costa Rica; 1992 [cited 2025 Apr 15]. https://docs.google.com/document/d/1k9HElPjANeSQFIXoZcyZe3o-tFoYS0mp9IFo-Dtie20/edit?pli=1&tab=t.0#:~:text=Capacidad%20de%20Carga%20Tur%C3%ADstica%20de%20las%20%C3%81reas%20de%20Uso%20P%C3%BAblico%20del%20Monumento%20Nacional%20Guayabo%2C%20Costa%20Rica. Accessed 15 Apr 2025.

[36] Jangra R, Kaushik SP. Estimating carrying capacity in a high mountainous tourist area: a destination conservation strategy. In: Singh R, editor. Global geographical heritage, geoparks and geotourism, advances in geographical and environmental sciences. Singapore: Springer Nature; 2021. p. 427–47.

[37] Joseph S, P M. Carrying capacity assessment in complex systems: A comprehensive revisit with enhanced elements. Atna J Tour Stud. 2024;19:171–200. 10.12727/ajts.32.8.

[38] Pouya S, Aghlmand M. Evaluation of urban green space per capita with new remote sensing and geographic information system techniques and the importance of urban green space during the COVID-19 pandemic. Environ Monit Assess. 2022. 10.1007/s10661-022-10298-z. PMC9361964 35922695

[39] Ministry of Housing and Urban Affairs. Urban and regional development plans formulation and implementation (URDPFI) guidelines [Internet]. 2014. https://mohua.gov.in/upload/uploadfiles/files/URDPFI%20Guidelines%20Vol%20I(2).pdf. Accessed 9 Apr 2025.

[40] Bardhan S, Sarkar S. Optimizing tourist flows through operative carrying capacity assessment: the case of Bakkhali coastal tourism, W.B., India. Sustain Soc Dev. 2024;2:2550. 10.54517/ssd.v2i3.2550.

[41] Hassanpour Kourandeh H, Fataei E. Estimation of tourism carrying capacity of Fandoqloo forest in Ardebil province, Iran. bulletin of environment, pharmacology and life sciences. 2013;2:64–70. Accessed 15 Apr 2025.

[42] Ajuhari Z, Aziz A, Yaakob SSN, Abu Bakar S, Mariapan M. Systematic literature review on methods of assessing carrying capacity in recreation and tourism destinations. Sustainability. 2023. 10.3390/su15043474.

[43] Census. India–Census of India - Uttarakhand - Series 06– Part XII B - District census handbook, Nainital [Internet]. 2001. https://censusindia.gov.in/nada/index.php/catalog/1317. Accessed 20 Jun 2025.

[44] United Nations. International recommendations for tourism statistics 2008 [Internet]. New York: United Nations; 2010 [cited 2025 May 2]. https://unstats.un.org/unsd/publication/Seriesm/SeriesM_83rev1e.pdf#page=21. Accessed 2 May 2025.

[45] SIUD RSTUAoA. Study of challenges faced by six towns of Uttarakhand–Report Nainital town. 2024.

[46] Survey of India. Naini Tāl guide map [Internet]. British Library. 1938 [cited 2025 May 2]. https://www.geographicus.com/P/AntiqueMap/Nainital-surveyofindia-1938. Accessed 2 May 2025.

[47] Murray J. A handbook for travellers in India, Burma, and Ceylon [Internet]. 1911 [cited 2025 May 2]. https://archive.org/details/handbooktravelle00john/handbooktravelle00john/page/n71/mode/2up?view=theater. Accessed 2 May 2025.

[48] Housing Department, Govt. of Uttarakhand. Building construction and development bye-laws, Uttarakhand [Internet]. 2019. https://cdnbbsr.s3waas.gov.in/s3ac1ad983e08ad3304a97e147f522747e/uploads/2025/01/20250104472272478.pdf. Accessed 9 Apr 2025.

[49] Himanshu Panwar. Proportion of green cover [Internet]. Rajasekar U, Shankar VTG, Kaur A, editors. Climate centre for cities, national institute of urban affairs; 2022 [cited 2025 Apr 15]. https://niua.in/c-cube/sites/all/themes/zap/assets/pdf/URBAN%20PLANNING/UPGCB2%20-%20GreenCover.pdf. Accessed 15 Apr 2025.

[50] Bhattacharyya P, DasGupta S, Das S, Paul S. Landslide susceptibility analysis: a case study of Nainital municipal area [Internet]. Res. Sq. 2020 [cited 2025 Apr 15]. 10.21203/rs.3.rs-106891/v1.

[51] CPCB. national inventory of sewage treatment plants central pollution control board Parivesh Bhawan east Arjun nagar , Delhi. 2021.

[52] Asian development bank. Uttarakhand integrated and resilient urban development project (RRP IND 38272–044) Initial environmental examination [Internet]. 2021. https://www.adb.org/projects/documents/ind-38272-044.

[53] Central public health and environmental engineering organisation (CPHEEO). Advisory on public and community toilets [Internet]. 2018 Nov. https://cpheeo.gov.in/upload/5d7f529ceed5eAdvisory%20on%20Public%20Toilet_compressed.pdf. Accessed 15 Apr 2025.

[54] Sati VP. The nature of tourism and tourists/pilgrims’ inflow in Uttarakhand Himalaya. J Multidiscip Acad Tour. 2020;5(2):115–24. 10.31822/jomat.731386.

[55] Wilcox R. Probability and related concepts. Applying contemporary statistical techniques [Internet]. Academic press; 2003 [cited 2025 Apr 15]. 10.1016/B978-012751541-0/50023-7. Accessed 15 Apr 2025.

[56] Shiraz Z. Nainital: Popular tourist spot Dorothy’s Seat collapses after landslide. Hindustan times. Nainital. 2024. https://www.hindustantimes.com/lifestyle/travel/nainital-popular-tourist-spot-dorothys-seat-collapses-after-landslide-101723113709955.html. Accessed 2 May 2025.

[57] The Telegraph. Landslide wipes away major portion of Nainital-Bhowali road in Uttarakhand. The Telegraph online [Internet]. Nainital; 2022 [cited 2025 May 2]; https://www.telegraphindia.com/india/landslide-wipes-away-major-portion-of-Nainital-bhowali-road-in-uttarakhand/cid/1877184. Accessed 2 May 2025.

[58] Hindustan Times. Landslide rocks Nainital lake area, boulders and debris fall into waters. Hindustan times [Internet]. Nainital; 2021 Aug [cited 2025 May 2]; https://www.hindustantimes.com/cities/dehradun-news/landslide-rocks-Nainital-lake-area-boulders-and-debris-fall-into-waters-101630412390981.html. Accessed 2 May 2025.

[59] Bhisht L. Nainital cut off from rest of Uttarakhand as torrential rain triggers landslides. India Today [Internet]. Nainital; 2021 [cited 2025 May 2]; https://www.indiatoday.in/india/story/uttarakhand-Nainital-rain-weather-news-flooding-pictures-videos-1866722-2021-10-19. Accessed 2 May 2025.

[60] Santoshi N. Fragile Nainital: Hillside survey planned after landslide, house collapse. Hindustan Times [Internet]. Nainital; 2023 Oct 23 [cited 2025 May 2]; https://www.hindustantimes.com/cities/dehradun-news/fragile-Nainital-hillside-survey-planned-after-landslide-house-collapse-101696252991973.html. Accessed 2 May 2025.

[61] Santoshi N. Landslides, road cave-ins trigger eco fears in Nainital. Hindustan Times [Internet]. Nainital; 2018 Sep 18 [cited 2025 May 2]; https://www.hindustantimes.com/dehradun/landslides-road-cave-ins-trigger-eco-fears-in-Nainital/story-rEyjHfHpDnSauFpPGHyoAO.html. Accessed 2 May 2025.

[62] SNS. Heavy snowfall in Uttarakhand’s Nainital triggers landslide in Naina peak. The statesman [Internet]. New Delhi; 2020 [cited 2025 May 2]; https://www.thestatesman.com/india/heavy-snowfall-in-uttarakhands-Nainital-triggers-landslide-in-naina-peak-1502850955.html. Accessed 2 May 2025.

[63] Ministry of urban development. Handbook of service level benchmarking [Internet]. 2011. https://cpheeo.gov.in/upload/uploadfiles/files/Handbook.pdf. Accessed 15 Apr 2025.

[64] Central ground water board department of water resources. National compilation on dynamic ground water resources of India [Internet]. 2024. https://cgwb.gov.in/cgwbpnm/public/uploads/documents/17357182991031590738file.pdf. Accessed 15 Apr 2025.

[65] Central pollution control board. The national action plan for municipal solid waste management [Internet]. 2015. https://cpcb.nic.in/uploads/MSW/Action_plan.pdf. Accessed 15 Apr 2025.

