# Supplementary material for: A framework for assessing urban carrying capacity and its impact on water and sanitation service delivery in a hill city
Source: Discov Sustain. 2026 May 12;7(1):1481. doi: 10.1007/s43621-026-02959-7 (PMC13550081; doi:10.1007/s43621-026-02959-7)
Supplement: Supplementary file 1 [file 43621_2026_2959_MOESM1_ESM.zip › 43621_2026_2959_MOESM1_ESM/R3_Revised_Appendix.docx]

**Appendix:**

1. **Table 1- Preliminary Criteria Catalogue:** All criteria pertaining to carrying capacity identified through literature review.

| **S.No.** | **Category** | **Indicator** | **Unit** | **Source** |
| --- | --- | --- | --- | --- |
| **1.** | **Water** | Per Capita water resource | m^3^ | [(Yang et al.,2010)](https://sci-hub.se/https:/doi.org/10.1016/j.cnsns.2009.09.027), [(Oh et al.,2005)](https://www.scribd.com/document/788762426/Determining-Development-Density-Using-the-Urban-Carrying-Capacity-Assessment-System), [(Wei et al.,2014)](https://www.semanticscholar.org/paper/Sustainable-urban-development%3A-A-review-on-urban-Wei-Huang/21cc7fa87507537893dbceef4cb07a64174ef0d5), [(Dong et al,2022)](https://www.mdpi.com/2071-1050/14/22/15124) |
|  |  | Per capita water supply | lpcd | [(Oh et al.,2005)](https://www.scribd.com/document/788762426/Determining-Development-Density-Using-the-Urban-Carrying-Capacity-Assessment-System), [(Bhagwat, 2021)](https://doi.org/10.1007/978-3-030-61891-9_6),  [(Schroll et al.,2012)](https://journal-tes.ruc.dk/wp-content/uploads/2021/05/no-5-Henning-Schroll.pdf) |
|  |  | Decrease of Water Consumption | GDP/Capita | [(Wei et al.,2014)](https://www.semanticscholar.org/paper/Sustainable-urban-development%3A-A-review-on-urban-Wei-Huang/21cc7fa87507537893dbceef4cb07a64174ef0d5) |
|  |  | Total amount of water resources | Hectares | [(Dong et al,2022)](https://www.mdpi.com/2071-1050/14/22/15124) |
|  |  | Drinking Water | lpcd | [Jangra and Kaushik (2021)](https://www.researchgate.net/publication/345320353_Estimating_Carrying_Capacity_in_a_High_Mountainous_Tourist_Area_A_Destination_Conservation_Strategy) |
|  |  | Water Consumption | m^3^ | [(Schroll et al.,2012)](https://journal-tes.ruc.dk/wp-content/uploads/2021/05/no-5-Henning-Schroll.pdf) |
|  |  | Water Quality, BOD levels | Mg/Litre | [(Schroll et al.,2012)](https://journal-tes.ruc.dk/wp-content/uploads/2021/05/no-5-Henning-Schroll.pdf) |
| **2.** | **Sanitation** | Percentage of sewage treatment | % | [(Yang et al.,2010)](https://sci-hub.se/https:/doi.org/10.1016/j.cnsns.2009.09.027),  [(Oh et al.,2005)](https://www.scribd.com/document/788762426/Determining-Development-Density-Using-the-Urban-Carrying-Capacity-Assessment-System) |
|  |  | Discharge intensity of COD per unit GDP | kg/unit GDP | [(Yang et al.,2010)](https://sci-hub.se/https:/doi.org/10.1016/j.cnsns.2009.09.027) |
|  |  | Ratio of reusable water reused | % | [(Wei et al.,2014)](https://www.semanticscholar.org/paper/Sustainable-urban-development%3A-A-review-on-urban-Wei-Huang/21cc7fa87507537893dbceef4cb07a64174ef0d5) |
|  |  | Recycling rate of industrial water | % | [(Wei et al.,2014)](https://www.semanticscholar.org/paper/Sustainable-urban-development%3A-A-review-on-urban-Wei-Huang/21cc7fa87507537893dbceef4cb07a64174ef0d5) |
|  |  | Urban industrial wastewater treatment rate | GDP/Capita | [(Wei et al.,2014)](https://www.semanticscholar.org/paper/Sustainable-urban-development%3A-A-review-on-urban-Wei-Huang/21cc7fa87507537893dbceef4cb07a64174ef0d5) |
|  |  | Density of drainage pipe in total built space | m^2^ | [(Wei et al.,2014)](https://www.semanticscholar.org/paper/Sustainable-urban-development%3A-A-review-on-urban-Wei-Huang/21cc7fa87507537893dbceef4cb07a64174ef0d5) |
|  |  | Wastewater Treatment Rate | % | [(Dong et al,2022)](https://www.mdpi.com/2071-1050/14/22/15124),  [(Schroll et al.,2012)](https://journal-tes.ruc.dk/wp-content/uploads/2021/05/no-5-Henning-Schroll.pdf) |
|  |  | Public bathrooms | # | [Jangra and Kaushik (2021)](https://www.researchgate.net/publication/345320353_Estimating_Carrying_Capacity_in_a_High_Mountainous_Tourist_Area_A_Destination_Conservation_Strategy) |
|  |  | Sewage Management | [-] | [(Bhagwat, 2021)](https://doi.org/10.1007/978-3-030-61891-9_6) |
|  |  | Wastewater Treatment Plants | # | [(Schroll et al.,2012)](https://journal-tes.ruc.dk/wp-content/uploads/2021/05/no-5-Henning-Schroll.pdf) |
| **3.** | **Solid Waste** | Waste treatment – composting, recycling | % | [(Oh et al.,2005)](https://www.scribd.com/document/788762426/Determining-Development-Density-Using-the-Urban-Carrying-Capacity-Assessment-System), [(Schroll et al.,2012)](https://journal-tes.ruc.dk/wp-content/uploads/2021/05/no-5-Henning-Schroll.pdf) |
|  |  | Rate of harmless disposal of domestic garbage | % | [(Wei et al.,2014)](https://www.semanticscholar.org/paper/Sustainable-urban-development%3A-A-review-on-urban-Wei-Huang/21cc7fa87507537893dbceef4cb07a64174ef0d5) |
|  |  | Volume of garbage disposal | tons | [(Dong et al,2022)](https://www.mdpi.com/2071-1050/14/22/15124) |
|  |  | Waste bins | # | [Jangra and Kaushik (2021)](https://www.researchgate.net/publication/345320353_Estimating_Carrying_Capacity_in_a_High_Mountainous_Tourist_Area_A_Destination_Conservation_Strategy) |
|  |  | Solid waste management | %/kg | [(Bhagwat, 2021)](https://doi.org/10.1007/978-3-030-61891-9_6) |
|  |  | Waste collection | %/kg | [(Schroll et al.,2012)](https://journal-tes.ruc.dk/wp-content/uploads/2021/05/no-5-Henning-Schroll.pdf) |
|  |  | Waste Segregation | %/kg | [(Schroll et al.,2012)](https://journal-tes.ruc.dk/wp-content/uploads/2021/05/no-5-Henning-Schroll.pdf) |
|  |  | Waste collection points, landfills | # |  |
| **4.** | **Environment and Ecology** | **Topography** | | |
|  |  | Elevation | m | [(Kourandeh et al.,2013)](https://bepls.com/nov_2013/10.pdf) [(Salemi et al., 2019)](https://doi.org/10.1080/13504509.2019.1570379) |
|  |  | Slope | Degrees (°) | [(](https://bepls.com/nov_2013/10.pdf)[Joseph et al., 2024)](https://journals.christuniversity.in/index.php/atna/article/view/4804), [(Kourandeh et al.,2013)](https://bepls.com/nov_2013/10.pdf), [(Salemi et al., 2019)](https://doi.org/10.1080/13504509.2019.1570379) ,  [(Siahaan et al.,2023)](https://www.cabidigitallibrary.org/doi/pdf/10.5555/20230433522) |
|  |  | Soil Texture |  | [(Salemi et al., 2019)](https://doi.org/10.1080/13504509.2019.1570379) |
|  |  | Soil Depth | m | [(Salemi et al., 2019)](https://doi.org/10.1080/13504509.2019.1570379) |
|  |  | Soil Erosion | m^2^ | [(Kourandeh et al.,2013)](https://bepls.com/nov_2013/10.pdf) [(](https://bepls.com/nov_2013/10.pdf)[Joseph et al., 2024)](https://journals.christuniversity.in/index.php/atna/article/view/4804), [(Salemi et al., 2019](https://doi.org/10.1080/13504509.2019.1570379) [(Siahaan et al.,2023)](https://www.cabidigitallibrary.org/doi/pdf/10.5555/20230433522) |
|  |  | Aspect | m | [(Salemi et al., 2019)](https://doi.org/10.1080/13504509.2019.1570379) |
|  |  | **Climate** | | |
|  |  | Temperature | °c | [Jangra and Kaushik (2021)](https://www.researchgate.net/publication/345320353_Estimating_Carrying_Capacity_in_a_High_Mountainous_Tourist_Area_A_Destination_Conservation_Strategy) |
|  |  | Rainfall | Days | [(Kourandeh et al.,2013)](https://bepls.com/nov_2013/10.pdf), [(Salemi et al., 2019)](https://doi.org/10.1080/13504509.2019.1570379), [(Siahaan et al.,2023)](https://www.cabidigitallibrary.org/doi/pdf/10.5555/20230433522), [(Bardhan and Sarkar,2024)](https://aber.apacsci.com/index.php/SSD/article/view/2550) |
|  |  | Snowfall | Days | [(](https://bepls.com/nov_2013/10.pdf)[Joseph et al., 2024)](https://journals.christuniversity.in/index.php/atna/article/view/4804), [(Kourandeh et al.,2013)](https://bepls.com/nov_2013/10.pdf) |
|  |  | Extreme Sunshine | Days | [(Kourandeh et al.,2013)](https://bepls.com/nov_2013/10.pdf)  [(Bardhan and Sarkar,2024)](https://aber.apacsci.com/index.php/SSD/article/view/2550),[(](https://bepls.com/nov_2013/10.pdf)[Joseph et al., 2024)](https://journals.christuniversity.in/index.php/atna/article/view/4804), [(Salemi et al., 2019)](https://doi.org/10.1080/13504509.2019.1570379) |
|  |  | **Green Cover** | | |
|  |  | Green Coverage Area / Vegetation | % | [(Yang et al.,2010)](https://sci-hub.se/https:/doi.org/10.1016/j.cnsns.2009.09.027), [(Oh et al.,2005)](https://www.scribd.com/document/788762426/Determining-Development-Density-Using-the-Urban-Carrying-Capacity-Assessment-System), [(Wei et al.,2014)](https://www.semanticscholar.org/paper/Sustainable-urban-development%3A-A-review-on-urban-Wei-Huang/21cc7fa87507537893dbceef4cb07a64174ef0d5),[(Dong et al,2022)](https://www.mdpi.com/2071-1050/14/22/15124),[(Kourandeh et al.,2013)](https://bepls.com/nov_2013/10.pdf), [(Siahaan et al.,2023)](https://www.cabidigitallibrary.org/doi/pdf/10.5555/20230433522) |
|  |  | Per capita public green area | m^2^ | [(Yang et al.,2010)](https://sci-hub.se/https:/doi.org/10.1016/j.cnsns.2009.09.027),  [(Wei et al.,2014)](https://www.semanticscholar.org/paper/Sustainable-urban-development%3A-A-review-on-urban-Wei-Huang/21cc7fa87507537893dbceef4cb07a64174ef0d5) |
|  |  | Coverage of City Forest | % | [(Wei et al.,2014)](https://www.semanticscholar.org/paper/Sustainable-urban-development%3A-A-review-on-urban-Wei-Huang/21cc7fa87507537893dbceef4cb07a64174ef0d5) |
|  |  | Area of Parks and Green land | Hectare | [(Dong et al,2022)](https://www.mdpi.com/2071-1050/14/22/15124) |
|  |  | **Air** | | |
|  |  | Energy | ppm/year | [(Oh et al.,2005)](https://www.scribd.com/document/788762426/Determining-Development-Density-Using-the-Urban-Carrying-Capacity-Assessment-System) |
|  |  | Air Quality | % | [(Yang et al.,2010)](https://sci-hub.se/https:/doi.org/10.1016/j.cnsns.2009.09.027) |
|  |  | Discharge intensity of SO2/GDP | Kg/1000 yuan | [(Yang et al.,2010)](https://sci-hub.se/https:/doi.org/10.1016/j.cnsns.2009.09.027) |
|  |  | **Disaster** | | |
|  |  | Landslide / Distance from Fault | m^2^ | [(](https://bepls.com/nov_2013/10.pdf)[Joseph et al., 2024)](https://journals.christuniversity.in/index.php/atna/article/view/4804) [(Salemi et al., 2019)](https://doi.org/10.1080/13504509.2019.1570379) |
|  |  | Flash Flood | m^2^ | [(](https://bepls.com/nov_2013/10.pdf)[Joseph et al., 2024)](https://journals.christuniversity.in/index.php/atna/article/view/4804) [(Salemi et al., 2019)](https://doi.org/10.1080/13504509.2019.1570379) |
|  |  | Flood / Distance from River | m^2^ | [(](https://bepls.com/nov_2013/10.pdf)[Joseph et al., 2024)](https://journals.christuniversity.in/index.php/atna/article/view/4804) |
|  |  | Cyclone/Typhoon | m^2^ | [(](https://bepls.com/nov_2013/10.pdf)[Joseph et al., 2024)](https://journals.christuniversity.in/index.php/atna/article/view/4804), [(Eduarte et al.,2021)](https://philjournalsci.dost.gov.ph/images/pdf/pjs_pdf/vol150no3/determination_of_carrying_capacity_estimates_of_ecotourism_attractions_.pdf) |
|  |  | Lighting and Thunder | m^2^ | [(](https://bepls.com/nov_2013/10.pdf)[Joseph et al., 2024)](https://journals.christuniversity.in/index.php/atna/article/view/4804) |
|  |  | Forest Fire | m^2^ | [(Kourandeh et al.,2013)](https://bepls.com/nov_2013/10.pdf) [(](https://bepls.com/nov_2013/10.pdf)[Joseph et al., 2024)](https://journals.christuniversity.in/index.php/atna/article/view/4804) |
| 5. | **Demography** | Population Density | Pop/sq.km | [(Yang et al.,2010)](https://sci-hub.se/https:/doi.org/10.1016/j.cnsns.2009.09.027) |
|  |  | Population Life Expectancy | % | [(Wei et al.,2014)](https://www.semanticscholar.org/paper/Sustainable-urban-development%3A-A-review-on-urban-Wei-Huang/21cc7fa87507537893dbceef4cb07a64174ef0d5) |
|  |  | Tourist Footfall | # | [(Dong et al,2022)](https://www.mdpi.com/2071-1050/14/22/15124), [Jangra and Kaushik (2021)](https://www.researchgate.net/publication/345320353_Estimating_Carrying_Capacity_in_a_High_Mountainous_Tourist_Area_A_Destination_Conservation_Strategy) |
|  |  | Tourism Density | T/ m^2^ | [Jangra and Kaushik (2021)](https://www.researchgate.net/publication/345320353_Estimating_Carrying_Capacity_in_a_High_Mountainous_Tourist_Area_A_Destination_Conservation_Strategy) |
|  |  | Ratio of Tourists to Residents | % | [(Dong et al,2022)](https://www.mdpi.com/2071-1050/14/22/15124),[(](https://bepls.com/nov_2013/10.pdf)[Joseph et al., 2024)](https://journals.christuniversity.in/index.php/atna/article/view/4804) |
|  |  | No. of students enrolled in universities |  |  |
| 6. | **Land use** | Plowland areas / Agricultural Land | m^2^ | [(Wei et al.,2014)](https://www.semanticscholar.org/paper/Sustainable-urban-development%3A-A-review-on-urban-Wei-Huang/21cc7fa87507537893dbceef4cb07a64174ef0d5), [(Salemi et al., 2019)](https://doi.org/10.1080/13504509.2019.1570379), [(Kourandeh et al.,2013)](https://bepls.com/nov_2013/10.pdf) |
|  |  | Constructive land per capita | m^2^ | [(Wei et al.,2014)](https://www.semanticscholar.org/paper/Sustainable-urban-development%3A-A-review-on-urban-Wei-Huang/21cc7fa87507537893dbceef4cb07a64174ef0d5) |
|  |  | Per capita urban land | m^2^ | [(Wei et al.,2014)](https://www.semanticscholar.org/paper/Sustainable-urban-development%3A-A-review-on-urban-Wei-Huang/21cc7fa87507537893dbceef4cb07a64174ef0d5) |
|  |  | Per capita floor space of houses in urban areas | m^2^ | [(Wei et al.,2014)](https://www.semanticscholar.org/paper/Sustainable-urban-development%3A-A-review-on-urban-Wei-Huang/21cc7fa87507537893dbceef4cb07a64174ef0d5) |
|  |  | Cultivated Land | Hectare | [(Dong et al,2022)](https://www.mdpi.com/2071-1050/14/22/15124) |
|  |  | Land for Construction | Hectare | [(Dong et al,2022)](https://www.mdpi.com/2071-1050/14/22/15124), [(](https://bepls.com/nov_2013/10.pdf)[Joseph et al., 2024)](https://journals.christuniversity.in/index.php/atna/article/view/4804) |
|  |  | Area available for Tourism | m^2^ | [Jangra and Kaushik (2021)](https://www.researchgate.net/publication/345320353_Estimating_Carrying_Capacity_in_a_High_Mountainous_Tourist_Area_A_Destination_Conservation_Strategy) |
|  |  | Area of watershed suitable for development | m^2^ | [(Bhagwat, 2021)](https://doi.org/10.1007/978-3-030-61891-9_6) |
|  |  | Area not suitable for development | m^2^ | [(Bhagwat, 2021)](https://doi.org/10.1007/978-3-030-61891-9_6) |
|  |  | Floor area ratio | [-] | [(Bhagwat, 2021)](https://doi.org/10.1007/978-3-030-61891-9_6) |
|  |  | Floor area requirement per head | m^2^ | [(Bhagwat, 2021)](https://doi.org/10.1007/978-3-030-61891-9_6) |
|  |  | Recreational Parks | m^2^ | [(Bhagwat, 2021)](https://doi.org/10.1007/978-3-030-61891-9_6) |
|  |  | Playgrounds | m^2^ | [(Bhagwat, 2021)](https://doi.org/10.1007/978-3-030-61891-9_6) |
|  |  | Open spaces | m^2^ | [(Bhagwat, 2021)](https://doi.org/10.1007/978-3-030-61891-9_6) |
|  |  | Rural Areas | m^2^ | [(Salemi et al., 2019)](https://doi.org/10.1080/13504509.2019.1570379) |
| 7. | **Urban Infrastructure** | Per capita area of paved road | m^2^ | [(Yang et al.,2010)](https://sci-hub.se/https:/doi.org/10.1016/j.cnsns.2009.09.027), [(Wei et al.,2014)](https://www.semanticscholar.org/paper/Sustainable-urban-development%3A-A-review-on-urban-Wei-Huang/21cc7fa87507537893dbceef4cb07a64174ef0d5) |
|  |  | Road / Length of Highways / Transportation/Mobility | Km | [(Dong et al,2022)](https://www.mdpi.com/2071-1050/14/22/15124), [(Wei et al.,2014)](https://www.semanticscholar.org/paper/Sustainable-urban-development%3A-A-review-on-urban-Wei-Huang/21cc7fa87507537893dbceef4cb07a64174ef0d5),[(Bhagwat, 2021)](https://doi.org/10.1007/978-3-030-61891-9_6),[(](https://bepls.com/nov_2013/10.pdf)[Joseph et al., 2024)](https://journals.christuniversity.in/index.php/atna/article/view/4804) |
|  |  | Subway System | m^2^ | [(Oh et al.,2005)](https://www.scribd.com/document/788762426/Determining-Development-Density-Using-the-Urban-Carrying-Capacity-Assessment-System) |
|  |  | Number of Hospital beds per thousand persons | # | [(Wei et al.,2014)](https://www.semanticscholar.org/paper/Sustainable-urban-development%3A-A-review-on-urban-Wei-Huang/21cc7fa87507537893dbceef4cb07a64174ef0d5),[(Bhagwat, 2021)](https://doi.org/10.1007/978-3-030-61891-9_6) |
|  |  | Total No. of Taxis/Cars | # | [(Dong et al,2022)](https://www.mdpi.com/2071-1050/14/22/15124) |
|  |  | Parking | # | [Jangra and Kaushik (2021)](https://www.researchgate.net/publication/345320353_Estimating_Carrying_Capacity_in_a_High_Mountainous_Tourist_Area_A_Destination_Conservation_Strategy) |
|  |  | Lighting | # | [Jangra and Kaushik (2021)](https://www.researchgate.net/publication/345320353_Estimating_Carrying_Capacity_in_a_High_Mountainous_Tourist_Area_A_Destination_Conservation_Strategy) |
|  |  | Food Joints | # | [Jangra and Kaushik (2021)](https://www.researchgate.net/publication/345320353_Estimating_Carrying_Capacity_in_a_High_Mountainous_Tourist_Area_A_Destination_Conservation_Strategy) |
|  |  | Power Supply | Hrs | [(Bhagwat, 2021)](https://doi.org/10.1007/978-3-030-61891-9_6) |
|  |  | Educational Institutes | #, Coverage rate | [(Bhagwat, 2021)](https://doi.org/10.1007/978-3-030-61891-9_6) |
|  |  | Socio-cultural facilities | #, Coverage rate | [(Bhagwat, 2021)](https://doi.org/10.1007/978-3-030-61891-9_6), [(Dong et al,2022)](https://www.mdpi.com/2071-1050/14/22/15124) |
|  |  | Communication post and telecom | Coverage rate | [(Bhagwat, 2021)](https://doi.org/10.1007/978-3-030-61891-9_6), [(Wei et al.,2015)](https://www.researchgate.net/profile/Yigang-Wei/publication/344576281_Using_Urban-Carrying_Capacity_as_a_Benchmark_for_Sustainable_Urban_Development_An_Empirical_Study_of_Beijing/links/5f8112fba6fdccfd7b554cb7/Using-Urban-Carrying-Capacity-as-a-Benchmark-for-Sustainable-Urban-Development-An-Empirical-Study-of-Beijing.pdf?origin=publication_detail&_tp=eyJjb250ZXh0Ijp7ImZpcnN0UGFnZSI6InB1YmxpY2F0aW9uIiwicGFnZSI6InB1YmxpY2F0aW9uRG93bmxvYWQiLCJwcmV2aW91c1BhZ2UiOiJwdWJsaWNhdGlvbiJ9fQ&__cf_chl_tk=ahneUJ.m5oR19ZKHuinsR3VEOLVe.8Cfc7ecoV3XsHE-1739353283-1.0.1.1-4xuD2K.ag9r3OQS3jX1PBJ5GSqhec5vs7OkhRXrpDc8),[(Salemi et al., 2019)](https://doi.org/10.1080/13504509.2019.1570379) |
|  |  | No. of public library/museum/park per capita | Coverage rate | [(Wei et al.,2015)](https://www.researchgate.net/profile/Yigang-Wei/publication/344576281_Using_Urban-Carrying_Capacity_as_a_Benchmark_for_Sustainable_Urban_Development_An_Empirical_Study_of_Beijing/links/5f8112fba6fdccfd7b554cb7/Using-Urban-Carrying-Capacity-as-a-Benchmark-for-Sustainable-Urban-Development-An-Empirical-Study-of-Beijing.pdf?origin=publication_detail&_tp=eyJjb250ZXh0Ijp7ImZpcnN0UGFnZSI6InB1YmxpY2F0aW9uIiwicGFnZSI6InB1YmxpY2F0aW9uRG93bmxvYWQiLCJwcmV2aW91c1BhZ2UiOiJwdWJsaWNhdGlvbiJ9fQ&__cf_chl_tk=ahneUJ.m5oR19ZKHuinsR3VEOLVe.8Cfc7ecoV3XsHE-1739353283-1.0.1.1-4xuD2K.ag9r3OQS3jX1PBJ5GSqhec5vs7OkhRXrpDc8) |
|  |  | Sports Facilities | Coverage rate | [(Wei et al.,2015)](https://www.researchgate.net/profile/Yigang-Wei/publication/344576281_Using_Urban-Carrying_Capacity_as_a_Benchmark_for_Sustainable_Urban_Development_An_Empirical_Study_of_Beijing/links/5f8112fba6fdccfd7b554cb7/Using-Urban-Carrying-Capacity-as-a-Benchmark-for-Sustainable-Urban-Development-An-Empirical-Study-of-Beijing.pdf?origin=publication_detail&_tp=eyJjb250ZXh0Ijp7ImZpcnN0UGFnZSI6InB1YmxpY2F0aW9uIiwicGFnZSI6InB1YmxpY2F0aW9uRG93bmxvYWQiLCJwcmV2aW91c1BhZ2UiOiJwdWJsaWNhdGlvbiJ9fQ&__cf_chl_tk=ahneUJ.m5oR19ZKHuinsR3VEOLVe.8Cfc7ecoV3XsHE-1739353283-1.0.1.1-4xuD2K.ag9r3OQS3jX1PBJ5GSqhec5vs7OkhRXrpDc8) |
|  |  | Hotels/Accommodation | # | [(](https://bepls.com/nov_2013/10.pdf)[Joseph et al., 2024)](https://journals.christuniversity.in/index.php/atna/article/view/4804) |
|  |  | Historical, Religious and Ancient Sites | m^2^ | [(Salemi et al., 2019)](https://doi.org/10.1080/13504509.2019.1570379) |
| 8. | **Social** | Accessibility | [-] | [Jangra and Kaushik (2021)](https://www.researchgate.net/publication/345320353_Estimating_Carrying_Capacity_in_a_High_Mountainous_Tourist_Area_A_Destination_Conservation_Strategy),[(Eduarte et al.,2021)](https://philjournalsci.dost.gov.ph/images/pdf/pjs_pdf/vol150no3/determination_of_carrying_capacity_estimates_of_ecotourism_attractions_.pdf) |
|  |  | Safety | [-] | [Jangra and Kaushik (2021)](https://www.researchgate.net/publication/345320353_Estimating_Carrying_Capacity_in_a_High_Mountainous_Tourist_Area_A_Destination_Conservation_Strategy), [(](https://bepls.com/nov_2013/10.pdf)[Joseph et al., 2024)](https://journals.christuniversity.in/index.php/atna/article/view/4804) |
|  |  | Animal Menace |  | [Jangra and Kaushik (2021)](https://www.researchgate.net/publication/345320353_Estimating_Carrying_Capacity_in_a_High_Mountainous_Tourist_Area_A_Destination_Conservation_Strategy), [(](https://bepls.com/nov_2013/10.pdf)[Joseph et al., 2024)](https://journals.christuniversity.in/index.php/atna/article/view/4804) |
|  |  | Seasonality |  | [(](https://bepls.com/nov_2013/10.pdf)[Joseph et al., 2024)](https://journals.christuniversity.in/index.php/atna/article/view/4804) |
|  |  | Strikes and Riots |  | [(Eduarte et al.,2021)](https://philjournalsci.dost.gov.ph/images/pdf/pjs_pdf/vol150no3/determination_of_carrying_capacity_estimates_of_ecotourism_attractions_.pdf) |
|  |  | Overcrowding |  | [(Eduarte et al.,2021)](https://philjournalsci.dost.gov.ph/images/pdf/pjs_pdf/vol150no3/determination_of_carrying_capacity_estimates_of_ecotourism_attractions_.pdf), [(](https://bepls.com/nov_2013/10.pdf)[Joseph et al., 2024)](https://journals.christuniversity.in/index.php/atna/article/view/4804) |
| 9. | **Economy** | Unemployment Rate | % | [(Wei et al.,2015)](https://www.researchgate.net/profile/Yigang-Wei/publication/344576281_Using_Urban-Carrying_Capacity_as_a_Benchmark_for_Sustainable_Urban_Development_An_Empirical_Study_of_Beijing/links/5f8112fba6fdccfd7b554cb7/Using-Urban-Carrying-Capacity-as-a-Benchmark-for-Sustainable-Urban-Development-An-Empirical-Study-of-Beijing.pdf?origin=publication_detail&_tp=eyJjb250ZXh0Ijp7ImZpcnN0UGFnZSI6InB1YmxpY2F0aW9uIiwicGFnZSI6InB1YmxpY2F0aW9uRG93bmxvYWQiLCJwcmV2aW91c1BhZ2UiOiJwdWJsaWNhdGlvbiJ9fQ&__cf_chl_tk=ahneUJ.m5oR19ZKHuinsR3VEOLVe.8Cfc7ecoV3XsHE-1739353283-1.0.1.1-4xuD2K.ag9r3OQS3jX1PBJ5GSqhec5vs7OkhRXrpDc8), [(Dong et al,2022)](https://www.mdpi.com/2071-1050/14/22/15124) |
|  |  | Per capita disposable income |  | [(Wei et al.,2015)](https://www.researchgate.net/profile/Yigang-Wei/publication/344576281_Using_Urban-Carrying_Capacity_as_a_Benchmark_for_Sustainable_Urban_Development_An_Empirical_Study_of_Beijing/links/5f8112fba6fdccfd7b554cb7/Using-Urban-Carrying-Capacity-as-a-Benchmark-for-Sustainable-Urban-Development-An-Empirical-Study-of-Beijing.pdf?origin=publication_detail&_tp=eyJjb250ZXh0Ijp7ImZpcnN0UGFnZSI6InB1YmxpY2F0aW9uIiwicGFnZSI6InB1YmxpY2F0aW9uRG93bmxvYWQiLCJwcmV2aW91c1BhZ2UiOiJwdWJsaWNhdGlvbiJ9fQ&__cf_chl_tk=ahneUJ.m5oR19ZKHuinsR3VEOLVe.8Cfc7ecoV3XsHE-1739353283-1.0.1.1-4xuD2K.ag9r3OQS3jX1PBJ5GSqhec5vs7OkhRXrpDc8) |
|  |  | Per capita GDP |  | [(Wei et al.,2015)](https://www.researchgate.net/profile/Yigang-Wei/publication/344576281_Using_Urban-Carrying_Capacity_as_a_Benchmark_for_Sustainable_Urban_Development_An_Empirical_Study_of_Beijing/links/5f8112fba6fdccfd7b554cb7/Using-Urban-Carrying-Capacity-as-a-Benchmark-for-Sustainable-Urban-Development-An-Empirical-Study-of-Beijing.pdf?origin=publication_detail&_tp=eyJjb250ZXh0Ijp7ImZpcnN0UGFnZSI6InB1YmxpY2F0aW9uIiwicGFnZSI6InB1YmxpY2F0aW9uRG93bmxvYWQiLCJwcmV2aW91c1BhZ2UiOiJwdWJsaWNhdGlvbiJ9fQ&__cf_chl_tk=ahneUJ.m5oR19ZKHuinsR3VEOLVe.8Cfc7ecoV3XsHE-1739353283-1.0.1.1-4xuD2K.ag9r3OQS3jX1PBJ5GSqhec5vs7OkhRXrpDc8) |
|  |  | Water consumption per unit GDP | Cu.m/unit GDP | [(Yang et al.,2010)](https://sci-hub.se/https:/doi.org/10.1016/j.cnsns.2009.09.027), [(Dong et al,2022)](https://www.mdpi.com/2071-1050/14/22/15124) |
|  |  | Energy consumption per 10,000 yuan of GDP | Tons of SCE | [(Dong et al,2022)](https://www.mdpi.com/2071-1050/14/22/15124) |
|  |  | Tourism revenue as a percentage of local GDP | % | [(Dong et al,2022)](https://www.mdpi.com/2071-1050/14/22/15124) |
|  |  | Foreign Exchange earnings from Tourism |  | [(Dong et al,2022)](https://www.mdpi.com/2071-1050/14/22/15124) |
| 10. | **Food Security** | Calorie Intake | Per capita/day | [(Schroll et al.,2012)](https://journal-tes.ruc.dk/wp-content/uploads/2021/05/no-5-Henning-Schroll.pdf) |
|  |  | Rice Yield | yield/hectare |  |
|  |  | On pesticides | Area |  |
|  |  | On nutrient levels and balance | Nitrogen ratio |  |

**2. Table 2- Primary list of Indicators**

| **Parameter** | **Indicator** | **Definition** | **Unit** |
| --- | --- | --- | --- |
| Water and Sanitation | Per capita water supply | The average amount of water received by each individual per day. | Lpcd |
|  | Extent of non-revenue water | The extent of NRW indicator signifies the extent of water produced which does not generate any revenue for the utility. | Mld |
|  | Water consumption (domestic, commercial and agricultural use) | The amount of water that is withdrawn from a source and consumed for domestic, commercial and agricultural purposes. | Mld |
|  | Continuity of water supplied | The continuity of water supply is the number of hours of supply provided to the households per day, standardized at 24 hours. | Hours/day |
|  | Water quality | Water quality is a measure of the suitability of water for a particular use based on physical, chemical, and biological characteristics. | [mg/l, #/ml] |
|  | Groundwater level | The depth or the level, either below ground or above the ordnance datum, at which soil or rock is saturated. | m |
|  | Surface water storage | The volume of freshwater stored in rivers/wetlands/floodplains/lakes( surface water bodies). | Ml |
|  | Wastewater generated | Wastewater generation refers to the volume of liquid waste that is generated by various sources, including residential, commercial and institutional establishments. | Mld |
|  | Coverage of  sewerage network | The total length of the sewerage network installed within the city boundary that facilitates the collection and disposal of sewage. | km |
|  | Untreated wastewater disposed | The volume of wastewater generated within the city boundary is disposed of without standard treatment. | mld |
|  | Wastewater  treatment rate (secondary and tertiary) | The proportion of wastewater volume treated against the total volume of wastewater received by the treatment facility. | % or MLD |
|  | Reused treated water | The amount of treated wastewater that is reused for different purposes such as agriculture, recharging water bodies etc. | % or MLD |
|  | Stormwater drainage coverage | Total length of stormwater drainage facilitating discharge of rainwater to avoid flooding. | km |
|  | Quality of sewage treatment | Quality of sewage treatment refers to how effectively a treatment process removes pollutants (like organic matter, solids, nutrients, and pathogens) to make wastewater safe for discharge or reuse. | [mg/l,#/ml] |
|  | No. of public toilets | No. of toilets available per square km in the city. | No./ km^2^ |
| Solid Waste | Household-level coverage of waste collection | No. of households facilitated by door-to-door waste collection in the city. | #Households |
|  | Disposal and recycling rate of solid waste | The amount of solid waste disposed and recycled against the total waste collected from households. | % |
|  | Provision of dustbins | No. of dustbins provided per sq. km in the city. | # |
|  | Extent of segregation of waste | Percentage of households practising waste segregation. | % |
|  | Waste disposal Site /dumping ground | Area of dumping yard or waste disposal site within the administrative boundaries of the city. | sq. km  (available within the city) |
| Demography | Population density | The number of people residing per square kilometre. | person/sq. km |
|  | Household density (ward-wise ) | The number of households per square kilometre | household/sq. km |
|  | Floating population | The population residing in the city for a certain amount of time for various reasons such as education, work, tourism etc. | persons |
|  | Tourist footfall  (peak season) | No. of tourists visiting the city in the peak season, also known as the high season, is the time of year when tourist attractions are most favourable due to weather or events and demand for travel-related services is highest. | persons |
|  | Annual tourist footfall | Total number of tourists who visit a particular destination or attraction over the year. This metric helps in assessing the popularity and economic impact of a location, as well as planning for resources, services, and infrastructure to accommodate visitors. | persons |
|  | Population with access to potable water | Population with access to safe drinking water. (In-house connection, private supply etc.) | Households |
|  | Population with access to toilets | Population with access to toilets with basic sanitation services. (Flush toilets, pour and flush toilets, pit latrines, community toilets) | % or # HH |
|  | Population affected by water-borne diseases | Population affected by illnesses caused by microscopic organisms, like viruses and bacteria, that are ingested through contaminated water or by coming in contact with faeces. (diarrhoeal diseases, cholera, shigella, typhoid, hepatitis A and E ) | % or # |
| Land use | Land use zoning (residential, commercial and tourism-allied commercial areas ) | Area or percentage of area demarcated for different land use such as residential, commercial and tourism allied commercial areas. | % or sq km |
|  | Tourism hotspots density | No. of tourist spots situated per sq km in the city. | # Tourist spots/  sq km |
|  | Built-up area | Area covered by buildings excluding green cover, barren land and water bodies. | Sq km |
|  | Green cover | Area except built-up and barren land. | Sq km |
|  | Protected green cover (reserved forest, protected forest, wildlife sanctuary, biosphere reserve etc.) | Green cover in the city which has been protected by the govt. authorities under the Forest Act 1927 and 1980. | Sq km |

**Table 3: Key Assessment Indicators**

| **S.No.** | **Parameter** | **Correction Factor** | **Unit** |
| --- | --- | --- | --- |
| 1. | Topography and Climate | Slope | Degrees (°) |
| 2. |  | Rainfall | Days |
| 3. |  | Temperature | °C |
| 4. | Ecologically Vulnerable Areas | Urban Green Cover | m^2^ |
| 5. |  | Landslide Prone Areas | m^2^ |
| 6. | Water Supply | Per capita water supply | lpcd |
| 7. |  | Maximum safe volume of the lake | m^3^ |
| 8. |  | Continuity of water supply | hrs |
| 9. |  | Annual rate of Groundwater extraction | per cent |
| 10. | Sanitation | Coverage of the sewerage network | per cent |
| 11. |  | Wastewater Treatment | MLD |
| 12. |  | Public Toilets | n |
| 13. | Solid waste management | Waste collection and recycling | tons/day |
| 14. |  | Provision of Dustbins | n |
| 15. | Tourism Facilities | Parking | n |
| 16. |  | Accommodation | n of beds |

**Table 4: Data Collection details**

| **Data Point** | **Source** | **Year** |
| --- | --- | --- |
| **Population** | [Census 2011](https://censusindia.gov.in/nada/index.php/catalog/1317/download/4282/DH_2011_0511_PART_B_DCHB_NAINITAL.pdf) | 2011 |
| **Municipal Area** | [Census 2011](https://censusindia.gov.in/nada/index.php/catalog/1317/download/4282/DH_2011_0511_PART_B_DCHB_NAINITAL.pdf) | 2011 |
| **Tourist Footfall (2000-2023)** | Tourism Department, SIUD, RSTUAoA | 2024 |
| **Reserve Forest Area** | KII with Municipal Official | 2024 |
| **Slope** | Digital Elevation Model - SRTM 1 Arc-second Global, (Latest data available on USGS Earth Explorer for Nainital) | 2014 |
| **Rainfall (1990-2023)** | NASA Power Database | 2023 |
| **Urban Green Cover** | Landsat 8-9, OLI/TRS C2 L2, USGS Earth Explorer | 2024 |
| **Landslide Prone areas** | Digital Elevation Model - SRTM 1 Arc-second Global, (Latest data available on USGS Earth Explorer for Nainital)  Landsat 8-9, OLI/TRS C2 L2, USGS Earth Explorer | 2014, 2024 |
| **Per capita supply** | KII with municipal official | 2024 |
| **Maximum safe volume of Naini lake (m3)** | KII with municipal official | 2024 |
| **Continuity of water supply (hrs.)** | KII with municipal official | 2024 |
| **Annual rate of groundwater extraction (%)** | [Central Ground Water Board Department of Water Resources](https://cgwb.gov.in/cgwbpnm/public/uploads/documents/17357182991031590738file.pdf) | 2024 |
| **Coverage of the sewerage network** | KII with municipal official | 2024 |
| **Wastewater**  **Treatment** | [Central Pollution Control Board](https://nrcd.nic.in/writereaddata/FileUpload/NewItem_210_Inventorization_of_Sewage-Treatment_Plant.pdf) | 2015, 2021 |
| **Waste collection and recycling** | KII with municipal official | 2024 |
| **Provisions of Dustbins** | KII with municipal official | 2024 |
| **Parking** | Secondary Data collected through municipal officials | 2024 |
| **Accommodation** | Secondary Data collected through municipal officials | 2024 |
| **Daily Tourist footfall** | KII with municipal official | 2024 |
| **No. of Public Toilets** | KII with municipal official | 2024 |
| **Water Abstraction** | Jal Sansathan, Nainital | 2024 |
| **No. of STPs** | [Central Pollution Control Board](https://nrcd.nic.in/writereaddata/FileUpload/NewItem_210_Inventorization_of_Sewage-Treatment_Plant.pdf) | 2021 |
| **Operational Capacity of STPs** | [Central Pollution Control Board](https://nrcd.nic.in/writereaddata/FileUpload/NewItem_210_Inventorization_of_Sewage-Treatment_Plant.pdf) | 2021 |
| **No. of Rainy days (2000-2024)** | NASA Power Database | 2024 |
| **No. of Landslide events (1997-2024)** | KII and ULB records  Literature - (Bhisht, 2021; Hindustan Times, 2021; Khanduri Sushil, 2019; Santoshi, 2018, 2023; Shiraz, 2024; SNS, 2020; The Telegraph, 2022) | 2024 |

2. **Pearson’s coefficient of correlation:**

3. **Definitions**

i. Limiting Magnitude of the Variable (ML)

ML refers to the part of a given variable that poses a constraint on the system’s capacity. It represents the magnitude of the variable that exceeds safe, acceptable, or functional thresholds for example, the area with slope greater than 30° that restricts development or use. ML is therefore the limiting component used to determine how much of the total system is unsuitable or constrained.

ii. Total Magnitude of the Variable (MT)

MT refers to the complete extent or total measurable magnitude of the variable within the study area. It represents the full baseline value such as the total municipal area of Nainital against which the limiting magnitude (ML) is compared to compute the correction factor.

iii. Real Management Capacity (Amc)

Real management capacity refers to the actual, observed, or existing level of management performance within the study area. It captures the real-world operational capacity after accounting for constraints such as resource shortages, infrastructure gaps, institutional limitations, or inefficiencies such as Nainital’s current per capita water supply of 112 lpcd. Amc therefore represents the practical management capability achieved on the ground.

iv. Ideal Management Capacity (Imc)

Ideal management capacity refers to the benchmarked, optimal, or standard level of management performance that should ideally be achieved under fully efficient, well-resourced, and uninterrupted conditions. It is typically derived from policy guidelines, service-level benchmarks, or best-practice norms such as the MoHUA benchmark of 135 lpcd for water supply. Imc represents the target capacity against which actual performance is evaluated.

4. **List of Assumptions**

- It is assumed that Non-Revenue Water (NRW) constitutes 15 percent of the total municipal water supply, accounting for standardized inefficiencies in the system.
- Based on Key Informant Interviews (KIIs), 95 percent of wastewater connections were identified; however, for analytical consistency, it is assumed that 100 percent of properties (including residential, commercial, and institutional establishments) are connected to the sewer network, due to the lack of disaggregated data for the remaining 5 percent.
- In accordance with the Central Public Health and Environmental Engineering Organization (CPHEEO) manual, it is assumed that 80 percent of the water supplied is converted into wastewater.
- The total capacity of operational Sewage Treatment Plants (STPs) in Nainital was obtained from the Central Pollution Control Board’s (CPCB) latest inventory on STP status.
- A safety margin of 15 percent is assumed for STP operations, implying that only 85 percent of the total installed treatment capacity is considered usable to account for routine maintenance and operational inefficiencies.
- Water demand for commercial and institutional establishments was calculated based on the Indian Standard IS 1172:1993 guidelines on water requirements for buildings.
- Residential water supply figures were determined through KIIs with municipal officials and other relevant stakeholders.
- It is assumed that wastewater generated from residential households is prioritized for treatment, as it constitutes a statutory obligation of the Urban Local Body (ULB). Only the residual treatment capacity is allocated for commercial and institutional wastewater.
- Daily water abstraction volumes were identified through KIIs conducted with relevant authorities.
- The distribution of supplied water is assumed to follow an 89 percent to 11 percent split between residential and commercial/institutional users, respectively.
- Population projections for the year 2025 were calculated using an arithmetic mean method, based on historical Census data.
- It is assumed that there are no secondary sources of water within the city limits, and that all water is sourced exclusively from municipal infrastructure.
- As per statutory service delivery norms, it is assumed that water supply to residential households is prioritized. Commercial and institutional supply is met only after fulfilling residential requirements.
- For tourism-related traffic estimations, it is assumed that each taxi carries an average of four passengers, based on common vehicle types (typically 5–7 seaters) operating in the region.
- It is assumed that hotel accommodation operates on a basis of double occupancy per room, with the provision of one additional bed per room.
- For sanitation infrastructure calculations, it is assumed that there is an equal distribution of male and female toilet facilities within each public toilet block.
- All data accessed from NASA Power House Database has been attributed with a 10 per cent error margin as stated by NASA across the database.

5. **Administrative Approval received from Urban Development Directorate (UDD), Government of Uttarakhand**
